# Health checks and cardiovascular risk factor values over six years’ follow-up: Matched cohort study using electronic health records in England

**DOI:** 10.1371/journal.pmed.1002863

**Published:** 2019-07-30

**Authors:** Samah Alageel, Martin C. Gulliford

**Affiliations:** 1 Community Health Sciences Department, College of Applied Medical Sciences, King Saud University, Riyadh, Kingdom of Saudi Arabia; 2 School of Population Health Sciences, Faculty of Life Sciences and Medicine, King’s College London, London, United Kingdom; Edinburgh University, UNITED KINGDOM

## Abstract

**Background:**

The National Health Service (NHS) in England introduced a population-wide programme for cardiovascular disease (CVD) prevention in 2009, known as NHS Health Checks. This research aimed to measure the cardiovascular risk management and cardiovascular risk factor outcomes of the health check programme during six years’ follow-up.

**Methods and findings:**

A controlled interrupted time series study was conducted. Participants were registered with general practices in the Clinical Practice Research Datalink (CPRD) in England and received health checks between 1 April 2010 and 31 December 2013. Control participants, who did not receive a health check, were matched for age, sex, and general practice. Outcomes were blood pressure, body mass index (BMI), smoking, and total cholesterol (TC) and high-density lipoprotein cholesterol (HDL). Analyses estimated the net effect of health check by year, allowing for the underlying trend in risk factor values and baseline differences between cases and controls, adjusting for age, sex, deprivation, and clustering by general practice. There were 127,891 health check participants and 322,910 matched controls. Compared with controls, health check participants had lower BMI (cases mean 27.0, SD 4.8; controls 27.3, SD 5.6, Kg/m^2^), systolic blood pressure (SBP) (cases 129.0, SD 14.3; controls 129.3, SD 15.0, mm Hg), and smoking (21% in health check participants versus 27% in controls), but total and HDL cholesterol were similar. Health check participants were more likely to receive weight management advice (adjusted hazard ratio [HR] 5.03, 4.98 to 5.08, *P* < 0.001), smoking cessation interventions (HR 3.20, 3.13 to 3.27, *P* < 0.001), or statins (HR 1.24, 1.21 to 1.27, *P* < 0.001). There were net reductions in risk factor values up to six years after the check for BMI (−0.30, −0.39 to −0.20 Kg/m^2^, *P* < 0.001), SBP (−1.43, −1.70 to −1.16 mm Hg, *P* < 0.001), and smoking (17% in health check participants versus 25% in controls; odds ratio 0.90, 0.87 to 0.94, *P* < 0.001). The main study limitation was that residual confounding may be present because randomisation was not employed; health check–associated measurement introduced differential recording that might cause bias.

**Conclusions:**

Our results suggest that people who take up a health check generally have lower risk factor values than controls and are more likely to receive risk factor interventions. Risk factor values show net reductions up to six years following a health check in BMI, blood pressure, and smoking, which may be of public health importance.

## Abstract 2

Samah Alageel and colleagues reveal that people who go for health checks generally have lower BMI, blood pressure and smoke less.

## Author summary

**Why was this study done?:**

- The National Health Service (NHS) in England introduced a population-wide programme for cardiovascular disease prevention in 2009, known as NHS Health Checks.
- A systematic review suggested that health checks do not lead to any reduction in morbidity and mortality either overall or from cardiovascular causes.
- Short-term evaluations of the health check programme identified small reductions in blood pressure, cholesterol levels, and body mass index (BMI), with a median of two years’ follow-up, but longer-term follow-up study is needed to evaluate the impact of the programme on risk factors and the provision of risk management interventions in primary care.

**What did the researchers do and find?:**

- We studied data from 127,891 participants who completed the health check and 322,910 matched controls over six years’ follow-up.
- People who attend the health check generally have lower cardiovascular risk factor levels than controls who did not attend the check
- Attending the health check was associated with increased delivery of risk factor management interventions and lower risk factor values over six years’ follow-up.

**What do these findings mean?:**

- Attending the health check was associated with reductions in cardiovascular disease risk factors sustained over six years, and the largest benefit was observed for reduction in smoking prevalence.
- Although risk management interventions were offered more frequently to health check participants, not all participants who had a health check and who were offered risk management participated in the interventions offered.
- There were differences in participant characteristics and risk factor recording between the health check and control group that may have biased the results of the current study.

## Background

Cardiovascular diseases (CVDs) account for one-third of global mortality [1], with 45% of all deaths in Europe being caused by CVDs [2]. CVD mortality rates have declined in the United States and United Kingdom, but almost 160,000 UK deaths and 610,000 US deaths annually are attributable to heart disease [3,4], and the declining trend in morality may now be stalling. Modifiable risk factors including increased blood pressure, smoking, elevated body mass index (BMI), alcohol misuse, low fruit and vegetable intake, physical inactivity, high blood glucose, and high cholesterol account for two-thirds of global CVD deaths [5]. The World Health Organisation and national governments have recommended policies to reduce the burden of CVD by implementing interventions aimed at improving CVD risk factors at the population and individual level [6].

In England, the National Health Service (NHS) Health Check programme for the primary prevention of CVD and related disorders was introduced in 2009 [7,8]. The programme is available to all individuals aged 40 to 74 years who have not been previously diagnosed of ischaemic heart disease, stroke, or diabetes and are not currently prescribed statins or antihypertensive drugs [7]. The health check estimates individuals’ risk of heart disease, stroke, type 2 diabetes, chronic kidney diseases, and some forms of dementia [7]. This is achieved by assessing individualised risk of developing CVD in the next 10 years [7]. In 2014, the health check mandated the use of QRISK2 for CVD risk assessment [9]. Personalised risk management interventions are provided to those at high risk. The NHS Health Check programme provides an opportunity to address multiple risk factors, which, if successful, might reduce the risk of several diseases.

The health check programme has proved controversial [10]. Introduction of the programme was supported by modelling estimates that suggested that the health check programme could prevent 1,600 heart attacks and strokes, at least 650 premature deaths, as well as 4,000 new cases of diabetes each year [11]. The estimated cost per quality adjusted life year (QALY) was approximately UK£3,000 [11]. Critics of the programme have pointed out that previous studies of health checks have demonstrated increased costs but no evidence of benefit [12]. The health check programme lacks standardisation [13], is mainly focused on risk factor detection [14], and is not linked to evidence-based intervention strategies. Uptake of the programme has been consistently lower than anticipated, with most areas showing less than 50% uptake [15]. There are important uptake inequalities with deprived populations and smokers showing lower uptake of checks [16].

Evaluation of the longer-term impact of health checks on CVD risk factor outcomes is needed. As the health check programme was introduced in 2010, long-term follow-up would provide further assessment of the programme’s effectiveness. Chang and colleagues [17] evaluated changes in risk factors with a median follow-up time of two years following the check using a difference-in-difference analysis and showed small reductions in BMI (−0.27 kg/m^2^), systolic blood pressure (SBP) (−2.51 mm Hg), and total cholesterol (TC) (−0.15 mmol/L). However, little is known about the possible longer-term outcomes of health checks. There is a need to examine the impact of the health check programme on the delivery of risk management interventions and quantify the impact of attending the health check on health outcomes [18] in order to evaluate the long-term impact of risk management interventions delivered in primary care. This research aimed to measure the risk management and risk factor outcomes of the health check programme up to six years following the health check and evaluate whether outcomes following the health check are more favourable than in controls who did not attend the health check.

## Methods

We conducted an interrupted-times series analysis with matched controls using the electronic health records of general practices contributing to the Clinical Practice Research Datalink (CPRD). The study protocol was granted scientific and ethical approval by the Medicine and Healthcare Regulatory Agency Independent Scientific Advisory Committee (ISAC: Protocol No. 16_085R). The study protocol is available at S1 Appendix. There were no changes to the analysis plan. The CPRD is one of the world’s largest primary care databases that includes prospectively collected, anonymised medical records from more than 700 general practices in the UK from 1990 to the present. The data set covers approximately 7% of the UK population and is considered to be representative of the UK population in terms of age, sex, and geographical distribution [19]. As 98% of the UK population is registered at a general practice, CPRD data can be considered to be population based [19]. This study is reported following the Strengthening the Reporting of Observational Studies in Epidemiology (STROBE) guideline (S1 Checklist).

The present study was restricted to general practices in England because the health check programme is only conducted in England. A cohort of participants who received the health check was compared with matched control participants. The health check cohort comprised all participants in England, who were aged 40 to 74 years and who had a health check recorded between 1 April 2010 and 31 December 2013. The occurrence of the health check was identified using Read medical codes indicating that a health check or CVD risk assessment was completed [20]. Consistent with the eligibility criteria for the NHS Heath Check, health check participants were excluded if they had diagnoses of ischaemic heart disease, stroke or diabetes, or were treated with antihypertensive drugs or statins before the date of the health check. The control cohort was sampled from the list of patients registered at the same CPRD general practices as health check patients. The same exclusion criteria were applied as for health check cases. From the remaining patients who met the eligibility criteria for a health check, up to four control participants were sampled by individual matching with health check participants on general practice, sex, and age (± two years). Fewer than four controls per health check case were obtained if there were insufficient eligible controls to meet the matching criteria, with 2.52 controls per case overall. The health check date of the matched case was employed as the index date for matched controls.

The final health check sample comprised 127,891 participants, from 431 general practices in England, with a health check recorded between 1 April 2010 and 31 December 2013 and 322,910 matched control participants who did not receive the check with follow-up data available up to the latest date of 31 March 2017 (sample selection is presented in S1 Fig). In the health check sample, 36,442 were matched with one control, 29,318 were matched with two controls, 20,692 were matched with three controls, and 41,439 were matched with four controls.

### Outcome measures and covariates

Outcome measures were CVD risk factor values recorded into CPRD electronic health records, including SBP (mm Hg), diastolic blood pressure (DBP; mm Hg), BMI (kg/m^2^), TC (mmol/L), high-density lipoprotein cholesterol (HDL; mmol/L), and smoking status. Glycated haemoglobin (HbA1c) data were recorded in only 21% of health check participants and 13% of controls and were not included in this analysis. Data on participants’ height, weight, BMI, and blood pressure measurements were extracted from participants’ additional files. When BMI was not recorded, values were derived from height and weight records when possible. Data on participants’ TC and HDL were extracted from participants’ test files. All TC recording using milligrams per decilitre (mg/dl) were converted to millimoles per litre (mmol/L). Records relating to smoking status were identified in participants’ clinical, referral, and additional files by searching Read codes relating to smoking and smoking advice. In addition, therapy records were searched for product codes indicating prescription of smoking cessation therapy [21]. Data on medication prescriptions for antihypertensive therapy (AHT) and statins were analysed. Antihypertensive medications included five groups: drugs acting on the rennin-angiotensin system (A), beta-blockers (B), calcium channel blockers (C), diuretics (D), and other antihypertensive drugs (O). Data on weight management and smoking cessation interventions were also analysed. Weight management interventions were classified into lifestyle advice (diet and physical activity), referrals for weight management services, and prescription of antiobesity drugs. Since 2010, Orlistat was the only antiobesity medication licensed in the UK. Smoking cessation interventions were divided into two categories: referrals to a smoking-cessation advisor or stop smoking clinic and medication (nicotine replacement therapy). To assess changes in CVD risk score following the health check, records of multiple risk factors (blood pressure, cholesterol/HDL ratio, BMI, and smoking) were required per participant per year. However, due to the amount of missing data, changes in CVD risk score was not included in this study, as only small proportion of the sample will have four risk factors recorded each year following the health check.

Covariates included age, sex, and fifth of deprivation. Participants’ age was defined at the index date. Social and material deprivation was classified using the English Index of Multiple Deprivation (IMD) 2015 [22]. IMD 2015 fifths linked via general practice postcode were used, as only a subset of participants had linked data for deprivation based on participant postcodes.

### Statistical analysis

Baseline risk factor values were evaluated using recording of risk factors from three years before the health check up to 30 days after the index date. The number of participants with missing records is presented for each risk factor outcome. Baseline values were evaluated in health check participants and controls and presented as proportions or mean and SD by sex. Risk factor values were compared between health check participants and controls using mean differences for continuous variables and odds ratios for categorical variables. These measures were estimated using the method of generalised estimating equations (GEE) to allow for the correlation of observations within each cluster of case and its matched controls (matched set) [23]. The association between the health check programme and CVD risk management interventions was evaluated using time-to-event analysis models, for which the event is the first recorded risk management intervention. For smoking-cessation interventions, smokers identified at baseline were included. In the time-to-event analysis model, the index date was the date of the first completed health check in the health check group, and their matched controls were assigned this date as the index date. Follow-up records were censored if a control participant received a health check after 31 December 2013.

An interrupted-time series (ITS) analysis was conducted to evaluate changes in risk factor values over time. Participant records were divided into one-year periods, from five years before the index date to a maximum of six years after. For participants with multiple recordings in one year, we used the mean of risk factor values per participant per year. The model employed was represented as follows [24]:

$$Y_{t}=\beta_{O}+\beta_{1}Year+\beta_{2}Group+\beta_{3}TimeAfter.$$

*Y_t_* represented the value of the risk factor in year *t*. The term *β*_1_*Year* represented the secular trend in risk factor values, as the annual increment in risk factor values, for both health check and control participants. The term *β*_2_*Group* represented the mean difference between the cases and controls during the entire study; this allows health check participants and control participants to have differing intercepts. [24] The term *β*_3_*TimeAfter* represents the change in slope for risk factor values following the health check. [24] As there was evident nonlinearity of effect following the health check, the term ‘TimeAfter’ was fitted as a factor with a separate value estimated for each year following the check. The coefficients for TimeAfter are the measures of association that are relevant to this study [24,25]. S1 Table presents the coding of terms in the ITS analysis.

As risk factor values were not recorded in every year for each participant, the method of last observation carried forward (LOCF) was used for up to three years following the last recorded value to maximise the data available for analysis [26]. As sensitivity analyses, we compared using LOCF for one year only and a complete case analysis. A model was fitted using the method of GEE to evaluate the effect of study group (health check or control), study year (−5 years to +6 years), and years following the health check as indicator variable for each post-health check effect [24]. Robust variance estimates were employed to adjust standard errors for clustering of observations (person years) by matched set. In order to control for potential confounders, covariates included deprivation indices, age, and sex because adjustment for matching variables is recommended to avoid possible bias [27]. The GEE method allowed for the correlation of observations by matched set [23]. In sensitivity analyses, allowing for clustering by general practice rather than matched set gave negligible differences in estimates and standard errors. As the sample size was large, *P* values were generally very small; the presentation emphasises estimation rather than hypothesis testing.

## Results

The analysis included 127,891 participants who received the health check between 1 April 2010 and 31 December 2013 and 322,910 matched controls (S1 Fig). The distribution of health check participants and controls by sex and deprivation fifth is shown in Table 1. A larger proportion of the sample were aged 45 to 54 years in both study groups than any other age group. Over half of the control sample (53%) were male, while about half of the health check sample were male (49%). Baseline risk factor values at the time of the health check are presented in Table 2. Mean BMI was lower at baseline in health check than control participants (cases mean 27.0, SD 4.8; controls 27.3, SD 5.6, Kg/m^2^), as were SBP (cases 129.0, SD 14.3; controls 129.3, SD 15.0, mm Hg) and DBP (cases 79.2, SD 8.7; controls 79.3, SD 9.2, mm Hg). A higher proportion of controls were smokers at the time of the health check compared to the health check sample (health check 21% versus controls 27%). These differences in CVD risk factor levels were based on participants with risk factor records up to the health check; a higher proportion of the controls did not have risk factors recorded compared with health check participants.

**Table 1:** Distribution of health check and control participants according to age at index date, sex, and index of multiple deprivation.

Variable	Category	Health check participants	Controls	Mean controls per case^1^
		Freq. (%)	Freq. (%)	
		(*n* = 127,891)	(*n* = 322,910)	
**Age group (years)**	40–44	19,250 (15.1)	61,625 (19.1)	2.98
	45–54	52,733 (41.2)	145,518 (45.1)	2.77
	55–64	38,406 (30.0)	84,467 (26.2)	2.24
	65–74	17,502 (13.7)	31,300 (9.7)	1.92
**Sex**	Male	63,188 (49.4)	170,965 (52.9)	2.71
**IMD 2015 quintile**	Least deprived	19,880 (15.5)	55,671 (17.2)	2.80
	2	29,724 (23.2)	71,179 (22.0)	2.39
	3	25,817 (20.2)	70,449 (21.8)	2.73
	4	24,729 (19.3)	57,846 (17.9)	2.34
	Most deprived	27,741 (21.7)	67,765 (20.9)	2.44

**Abbreviations:** IMD, Index of Multiple Deprivation.

^1^ Mean number of controls matched for each health check participant.

**Table 2:** Risk factor values at the time of the health check in the health check and control groups.

		Health check^a^		Control^b^
	Freq.	(Total = 127,891)	Freq.	(Total = 322,910)
BMI (Kg/m^2^), mean (SD)	124,506	27.0 (4.8)	134,250	27.3 (5.6)
SBP (mm Hg), mean (SD)	126,753	129.0 (14.3)	188,435	129.3 (15.0)
DBP (mm Hg), mean (SD)	126,753	79.2 (8.7)	188,435	79.3 (9.2)
Total cholesterol (mmol/l), mean (SD)	121,796	5.43 (0.99)	85,019	5.47 (0.96)
HDL cholesterol (mmol/l), mean (SD)	115,038	1.48 (0.43)	75,770	1.46 (0.42)
Current smoking, ***n*** (%)	127,599	26,816 (21)	309,647	84,287 (27)

**Abbreviations:** BMI, body mass index; CI, confidence interval; CVD, cardiovascular disease; DBP, diastolic blood pressure; HDL, high-density lipoprotein; OR, odds ratio; SBP, systolic blood pressure.

^a^ Values from three years before the check and up to 30 days after the check.

^b^ Using generalised estimating equations, differences are values for cases minus values for controls; ORs contrast proportion for cases compared with controls.

**Fig 1:**
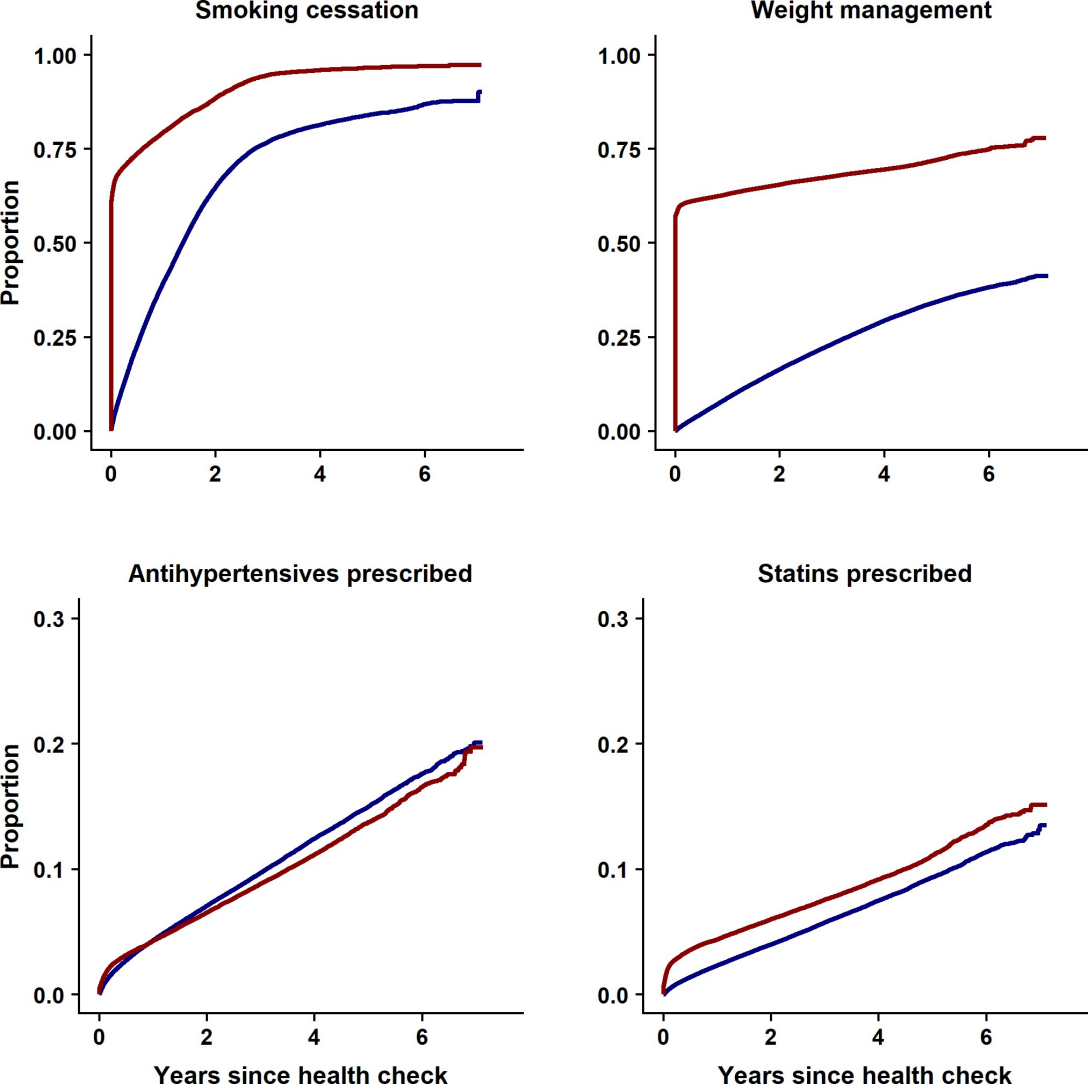
Results of time-to-event analysis showing risk management interventions offered in health check and control groups from the index date to the end of six years’ study follow-up.

Differences in the provision of risk management interventions between health check and control groups over six years’ follow-up are presented in Fig 1 and Table 3. For medication prescription, the health check programme was associated with a 24% relative increase in statin prescription, but AHT was prescribed less in the health check group than the controls (hazard ratio [HR] 0.86; 0.85 to 0.88, *P* < 0.001). The provision of weight management interventions was higher in the health check group than in the control group (HR 5.03; 4.98 to 5.08, *P* < 0.001). Smoking-cessation interventions were offered more often to smokers in the health check group than to smokers in the control group (HR 3.20; 3.13 to 3.27, *P* < 0.001). There was a strong evidence of nonproportional hazard for the provision of risk management interventions, as the majority were offered in the first year of follow-up as a result of the health check, and HRs represent the average effect over the period.

**Table 3:** Provision of risk management interventions to health check and control groups over six years’ follow-up.

Risk management interventions		Health check participants	Controls	HR^a^
		Freq. (%)	Freq. (%)	(95% CI, *P* value)
**Medications**	Statins	9,948 (8)	16,204 (5)	1.24 (1.21 to 1.27, <0.001)
	Antihypertensive drugs	11,633 (9)	27,324 (8)	0.86 (0.85 to 0.88, <0.001)
**Weight management interventions**	All interventions	86,412 (68)	64,711 (20)	5.03 (4.98 to 5.08, <0.001)
	Advice	85,124 (67)	61,917 (19)	2.46 (2.38 to 2.54, <0.001)
	Referrals	7,165 (6)	6,461 (2)	5.11 (5.06 to 5.16, <0.001)
	Medication	897 (0.7)	1,492 (0.5)	1.34 (1.23 to 1.45, <0.001)
**Smoking cessation interventions**	All interventions	19,927 (91)	49,282 (61)	3.20 (3.13 to 3.27, <0.001)
	Referrals	19,818 (90)	48,900 (61)	3.13 (3.07 to 3.20, <0.001)
	Medication	3,956 (18)	8,630 (11)	1.63 (1.57 to 1.69, <0.001)

**Abbreviations:** CI, confidence interval; HR, hazard ratio.

^a^Adjusted for age, sex, and deprivation quintile; HRs represent the average effect over the period of follow-up.

**Fig 2:**
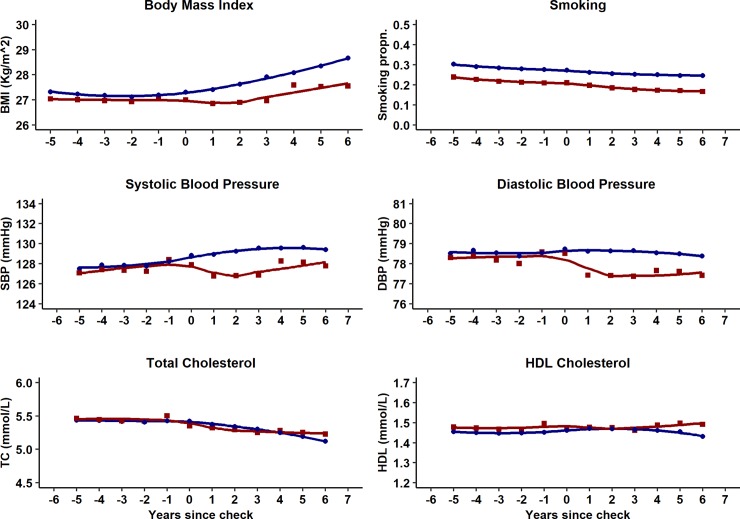
Risk factor trajectories of health check and control groups five years before and six years after the index date.

**Fig 3:**
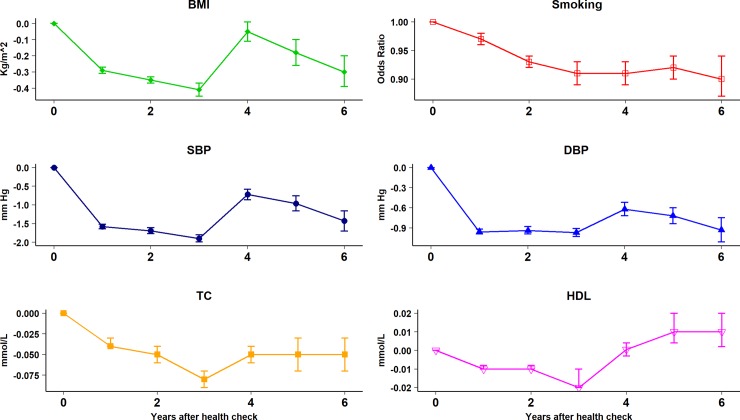
Net risk factor trajectories six years after the health check. Estimates are mean differences (95% confidence interval) between cases and controls adjusted for baseline differences, underlying trend, age, sex, deprivation fifth, and matched set.

S2 Fig presents proportions of participants with risk factor recorded by year during the study period. Risk factor recording was generally similar for health check participants and controls before the check but recording increased following the check for BMI, blood pressure, TC, and HDL cholesterol values. Fig 2 presents risk factor trajectories over time following the health check. Table 4 and Fig 3 present adjusted estimates from the ITS analysis for baseline differences, trends over time, and net changes following the health check for health check participants and controls over the study period. Overall mean BMI was lower in the health check group (0.27, 95% confidence interval 0.23 to 0.31 kg/m^2^, *P* < 0.001) compared to the control group, with an increasing trend in BMI over time (0.08, 0.07 to 0.09 kg/m^2^ per year, *P* < 0.001). After six years’ follow-up, the mean decrement in BMI following a health check was 0.30 kg/m^2^ (0.20 to 0.39 Kg/m^2^, *P* < 0.001). Over the study period, smoking decreased in both health check and control groups, with a reduction in odds of smoking of 3% per year (OR 0.97, 0.96 to 0.97 per year, *P* < 0.001). Health check participants were less likely to be smokers than controls after six years’ follow-up (health check 17% versus controls 25%; OR 0.90, 0.87 to 0.94, *P* < 0.001). Mean SBP (−1.21, −1.29 to −1.13 mm Hg, *P* < 0.001) and mean DBP (−0.45, −0.51 to −0.39 mm Hg, *P* < 0.001) were lower overall in the health check group compared to the control group. After six years’ follow-up, the mean decrement following a health check was 1.43 (1.16 to 1.70, *P* < 0.001) mm Hg for SBP and 0.93 (0.75 to −1.11 mm Hg, *P* < 0.001) for DBP compared to controls. Mean TC (0.01, 0.002 to 0.02 mmol/L, *P* < 0.001) and mean HDL (0.01, 0.006 to 0.01 mmol/L, *P* < 0.001) were slightly higher in the health check group compared to the control group. After six years’ follow-up, mean TC was 0.05 (0.03 to 0.07, *P* < 0.001) mmol/L lower after the health check. Mean HDL cholesterol was 0.01 (0.002 to 0.02, *P* < 0.001) mmol/L higher six years after the health check. During the first three years following the health check, there was a higher proportion of CVD risk factors recorded in health check participants than in controls (Fig 2). This might explain part of the reduction in CVD risk factor values, as low risk participants are more likely to have a risk factor recorded in health check group than controls.

**Table 4:** ITS analysis comparing health check and control participants. Figures are adjusted mean differences (95% CI, *P* value) except where indicated.

	Mean difference between cases and controls	Mean change per year for cases and controls	Year following the health check					
			1^st^ year	2^nd^ year	3^rd^ year	4^th^ year	5^th^ year	6^th^ year
BMI, mean, Kg/m^2^	−0.27	0.08	−0.29	−0.35	−0.41	−0.05	−0.18	−0.30
	(-0.31 to -0.23, <0.001)	(0.07 to 0.09, <0.001)	(-0.31 to -0.27, <0.001)	(-0.37 to -0.33, <0.001)	(-0.45 to -0.37, <0.001)	(-0.11 to 0.01, 0.07)	(-0.26 to -0.10, <0.001)	(-0.39 to -0.20, <0.001)
Current smoking, OR	0.70	0.97	0.97	0.93	0.91	0.91	0.92	0.90
	(0.69 to 0.71, <0.001)	(0.96 to 0.97, <0.001)	(0.96 to 0.98, <0.001)	(0.92 to 0.94, <0.001)	(0.89 to 0.93, <0.001)	(0.89 to 0.93, <0.001)	(0.90 to 0.94, <0.001)	(0.87 to 0.94, <0.001)
SBP, mean, mm Hg	−1.21	0.18	−1.58	−1.69	−1.90	−0.72	−0.96	−1.43
	(−1.29 to −1.13, <0.001)	(0.16 to 0.20, <0.001)	(−1.64 to −1.52, <0.001)	(−1.77 to −1.61, <0.001)	(−1.99 to −1.80, <0.001)	(−0.86 to −0.58, <0.001)	(−1.16 to −0.76, <0.001)	(−1.70 to −1.16, <0.001)
DBP, mean, mm Hg	−0.45	−0.04	−0.96	−0.94	−0.97	−0.62	−0.72	−0.93
	(−0.51 to −0.39, <0.001)	(−0.05 to −0.03, <0.001)	(−0.99 to −0.92, <0.001)	(−0.99 to −0.88, <0.001)	(−1.03 to −0.91, <0.001)	(−0.72 to −0.52, <0.001)	(−0.84 to −0.60, <0.001)	(−1.11 to −0.75, <0.001)
TC, mean, mmol/L	0.01	−0.02	−0.04	−0.05	−0.08	−0.05	−0.05	−0.05
	(0.002 to 0.02, 0.06)	(−0.02 to −0.01, <0.001)	(−0.04 to −0.03, <0.001)	(−0.06 to −0.04, <0.001)	(−0.09 to −0.07, <0.001)	(−0.06 to −0.04, <0.001)	(−0.07 to −0.03, <0.001)	(−0.07 to −0.03, <0.001)
HDL cholesterol, mean, mmol/L	0.01	0.004	−0.01	−0.01	−0.02	0.0005	0.01	0.01
	(0.006 to 0.01, <0.001)	(0.003 to 0.005, <0.001)	(−0.01 to −0.008, <0.001)	(−0.01 to −0.008, <0.001)	(−0.02 to −0.01, <0.001)	(−0.003 to 0.004, 0.83)	(0.004 to 0.02, 0.01)	(0.002 to 0.02, 0.21)

**Abbreviations:** BMI, body mass index; CI, confidence interval; DBP, diastolic blood pressure; HDL, high-density lipoprotein; OR, odds ratio; SBP, systolic blood pressure, TC, total cholesterol.

Differences were estimated as case controls using generalised estimation equation models, adjusting for each variable shown as well as age, sex, and deprivation fifth.

### Sensitivity analyses

Changes in CVD risk factor values estimated separately by sex are presented in S2 Table. The analysis showed only small differences in CVD outcomes between male and female participants with slightly greater reductions in blood pressure outcomes in female participants six years following the check compared to male participants. Analyses were repeated using the method of last observation carried forward for one year only, and this showed small differences in estimated CVD risk factors outcomes, compared with the primary analysis and results are presented in S3 Table. A further analysis was conducted using recorded data only and shown large proportion of missing data (S3 Fig) and smaller changes following the health check (S4 Table).

The number of controls per health check case differed according to levels of covariates (Table 1). Therefore, we conducted a sensitivity analysis including only one control per case. The results are shown in S5 Table; there were only slight differences in estimates and confidence intervals and no material difference in interpretation.

## Discussion

A controlled ITS study was conducted to measure CVD risk management and CVD risk factor outcomes of the health check programme during six years’ follow-up. This study shows that health check participants had slightly more favourable CVD risk factor values at the time of the health check compared with controls. In particular, the frequency of current smoking was 6% lower in health check attenders than controls. We observed that a key effect of the health check programme is an increase in provision of risk management advice. One additional patient received weight management intervention for every two patients receiving health checks; one additional patient received smoking cessation interventions for every three health cheeks performed in smokers; one additional patient was prescribed statins for every 33 health checks performed. After the health check, there were net reductions in BMI, SBP, and current smoking. At the end of six-years’ follow-up, the mean BMI was 0.3 Kg/m^2^ lower, and the odds of smoking were 10% lower in health check participants than controls at the end of study follow-up. The health check programme did not appear to be associated with relevant changes in TC and HDL levels. While net changes in risk factor values were generally of small magnitude, these were sustained for up to six years following the health check, and the cumulative impact of these changes could be of public health importance across the population at risk [28]. The health check programme aims to recruit the entire population of 40 to 74 years olds into a five-yearly cycle of checks, but interventions are primarily delivered to individuals who are found to be at high risk. Benefits will be proportionately larger if the high-risk population is considered as the denominator. This study did not evaluate the cost-effectiveness of the health check programme nor the impact on inequalities in cardiovascular risk.

There is limited evidence on the evaluation of CVD risk assessment and management programmes in routine care settings. Several systematic reviews have measured the impact of risk assessment and management interventions in primary prevention populations using randomised controlled trials [12,29,30], including the Oxford and Collaborators Health Check trial [31] and the British Family Heart Study [32]. These meta-analyses suggested that health checks in primary care are associated with small improvements in risk factors and no impact on total mortality (risk ratio 0.99; 0.95 to 1.03) [12], CVD mortality (risk ratio 1.03; 0.91 to 1.17) [12], or morbidity [12,29]. Our findings show that the outcomes of the health check programme may be generally consistent with those of randomised controlled trials in primary care with improvements in CVD risk factors over longer-term follow-up. Previous evaluation of the health check programme suggested slightly larger reductions in CVD risk factors with a median of two years’ follow-up [17] compared to our findings of six years’ follow up. Moreover, the current study evaluated the impact of the programme using ITS methods, thus taking into consideration changes in CVD risk factors prior to the health check.

The current study suggests reductions in smoking proportions six years following the health check. Smokers in the health check group were considerably more likely to be prescribed smoking-cessation medication than smokers in the control group. In their systematic review of multiple risk factor interventions in primary care, Alageel and colleagues [30] found only two trials reported offering smoking-cessation medications as part of their intervention. Nicotine replacement therapy is considered an effective strategy in sustaining smoking cessation, even among smokers who are not ready to quit smoking. [33,34]

The health check was also associated with increased provision of risk management interventions in the form of behaviour change advice and referrals. The increased provision of risk management interventions in the health check programme was associated with more limited improvements in CVD risk factors than for smoking. A recent systematic review of patients’ experience with the health check identified several challenges in relation to risk management intervention component [35]. Health check participants reported receiving behaviour change interventions, but many considered that messages were generic and lacked detail. Some participants would have liked a more proactive approach from their healthcare provider in supporting them to make behaviour change and felt they needed long-term follow-up and monitoring. [35] Understanding factors associated with the implementation of risk management interventions in primary care is essential to understand intervention outcomes. In a previous study, we found that healthcare professionals lacked guidance on how to implement risk management interventions and that implementation was restricted by the lack of time and follow-up in primary care. [36] Healthcare professionals’ doubts about the effectiveness of the interventions also affected their implementation [36,37]. There is a need to develop and implement effective, brief behaviour change techniques to facilitate risk factor management in primary care.

### Strengths and limitations

The CPRD is a large data set and it is broadly representative of the UK population, and the study findings may be generalisable to the wider population. The study employed a large sample of 450,801 individuals from 431 general practices in England, enabling precise estimation of effects. A high level of ascertainment of the health check was expected because use of mandated Read codes is required for reimbursement of practices. A randomised controlled trial would provide the optimal study design for evaluation, but the health check programme is a national policy. The present nonrandomised design offered the highest achievable level of evidence with the interrupted time series analysis being more resistant to bias than other nonrandomised designs. [38] We matched participants on major non-modifiable factors including age, sex, and general practice, but in a nonrandomised study, there is a concern for residual confounding from unmeasured variables including genetic and environmental variables. There were differences in risk factor recording and risk factor values between the health check and control group that may have biased the results of the current study. We found that baseline cardiovascular risk factor values were generally more favourable in health check participants, which indicates selection bias operating at the level of health check uptake. This is consistent with concerns that the health check programme might increase inequalities if the offer of a check is taken up by healthier individuals. Previous evidence has suggested lower uptake of health checks among groups at greatest risk of CVD [39,40], and health checks performed opportunistically reach higher risk participants compared with individuals responding to the health check invitation [39,41]. We also note that eligibility criteria might have been ascertained more completely for cases, who received a health check, than for controls, who did not receive a health check, leading to possible bias from differential misclassification. We employed a matched design but an unmatched analysis of the entire cohort, with health check as time-varying exposure, would provide an alternative approach to analysis. This was not feasible because of the very large resulting sample size.

A general limitation of research using electronic records is the level of missing data, with measurements often being recorded only when clinically indicated [42]. We addressed missing values by adopting a LOCF strategy. The direction of bias from differential recording may be difficult to anticipate, but missing values might have introduced bias in favour of the health check [26] if control participants were more likely to have values recorded that were high, while attendance at the health check might extend testing to a lower-risk section of the population. Brief advice may be given to participants but not recorded by clinicians, which could have led to underestimation of intervention rates in the control group. This is less likely to be an issue with referrals and drug prescribing. However, recording of risk management prescriptions and referrals may not necessarily results in participants dispensing medications or taking on behaviour change services referrals.

### Conclusions

Results from this study suggest that health check participants had slightly more favourable CVD risk factor values at the time of the health check compared with controls. Engagement in the health check programme was associated with increased provision of weight management and smoking-cessation advice and prescription of statins. There were net reductions in BMI, SBP, and current smoking, which were sustained for up to six years following the health check, and these could be of public health importance across the population at risk [28]. We caution that the lack of randomisation and the frequency of missing values might have contributed to bias.

## Supporting information

S1 Checklist STROBE checklist. STROBE, Strengthening the Reporting of Observational Studies in Epidemiology.
(DOC)

S1 Appendix Study protocol. (DOC)

S1 Fig Sample selection flow chart. (DOCX)

S2 Fig Proportion of participants with risk factor values recorded by year. Red, health check participants; blue, control participants.
(DOCX)

S3 Fig Risk factors trajectories of health check and control groups five years before and six years after the index date and proportion of participants with risk factor recorded throughout the study period for health check and control groups. (DOCX)

S1 Table Coding of interrupted time series analysis terms. (DOCX)

S2 Table Regression analysis of the association of the NHS Health Check programme with changes in risk factors means, by sex. NHS, National Health Services.
(DOCX)

S3 Table Interrupted time series analysis of one year carried forward comparing health check and control participants. Figures are adjusted mean differences (95% confidence interval) except where indicated.
(DOCX)

S4 Table ITS analysis of recorded data comparing health check and control participants. Figures are adjusted mean differences (95% confidence interval) except where indicated. ITS, interrupted-time series.
(DOCX)

S5 Table ITS analysis comparing health check and control participants. Sensitivity analysis with one control per case. Figures are adjusted mean differences (95% confidence interval) except where indicated. ITS, interrupted-time series.
(DOCX)

## Abbreviations

AHT: antihypertensive therapy
BMI: body mass index
CPRD: Clinical Practice Research Datalink
CVD: cardiovascular disease
DBP: diastolic blood pressure
GEE: generalised estimating equations
HbA1c: glycated haemoglobin
HDL: high-density lipoprotein cholesterol
HR: hazard ratio
IMD: Index of Multiple Deprivation
ITS: interrupted-time series
LOCF: last observation carried forward
NHS: National Health Service
QALY: quality adjusted life year
SBP: systolic blood pressure
TC: total cholesterol
