# Supplementary material for: Health checks and cardiovascular risk factor values over six years’ follow-up: Matched cohort study using electronic health records in England
Source: PLoS Med. 2019 Jul 30;16(7):e1002863. doi: 10.1371/journal.pmed.1002863 (PMC6667114; doi:10.1371/journal.pmed.1002863)
Supplement: S1 Checklist — STROBE, Strengthening the Reporting of Observational Studies in Epidemiology. (DOC) [file pmed.1002863.s001.doc]

# ISAC APPLICATION FORM

# PROTOCOLS FOR RESEARCH USING THE CLINICAL PRACTICE RESEARCH DATALINK (CPRD)

| ISAC use only:  Protocol Number  Date submitted | .............................  ............................. | **IMPORTANT**  **If you have any queries, please contact ISAC Secretariat:** ISAC[@cprd.com](mailto:Annalisa.Rubino@gprd.com) |
| --- | --- | --- |

| **Section A: The study** | | |
| --- | --- | --- |
| 1. **Study Title**   **Long-term outcomes of NHS Health Checks in England.** | | |
| 1. **Has any part of this research proposal or a related proposal been previously submitted to ISAC?**   Yes No  *If Yes, please provide previous protocol numbers*: 13- 071A | | |
| 1. **Has this protocol been peer reviewed by another Committee? (e.g. grant award or ethics committee)**   Yes No  *If Yes, please state the name of the reviewing Committee(s) and provide an outline of the review process and outcome:* | | |
| 1. **Type of Study** (please tick all the relevant boxes which apply)   Adverse Drug Reaction/Drug Safety Drug Utilisation  Disease Epidemiology  Drug Effectiveness  Pharmacoeconomics  Methodological  Health/Public Health Services Research  Post-authorisation Safety  **Other^*^**  ^*^Please specify the type of study in the lay summary | | |
| 1. **This study is intended for** (please tick all the relevant boxes which apply)**:**   Publication in peer reviewed journals  Presentation at scientific conference  Presentation at company/institutional meetings  Regulatory purposes  Other | | |
| **Section B: The Investigators** | | |
| 1. **Chief Investigator** (full name, job title, organisation name & e-mail address for correspondence- see guidance notes for eligibility)   Martin Gulliford, Professor of Public Health, King’s College London martin.gulliford@kcl.ac.uk  CV has been previously submitted to ISAC  **CV number:** 150_15CESL  A new CV is being submitted with this protocol  An updated CV is being submitted with this protocol | | |
| 1. **Affiliation** (full address)   King’s College London, Department of Primary Care and Public Health Sciences, Addison House Guy’s Campus London SE1 1UL | | |
| 1. **Corresponding Applicant**   Samah Alageel, Research Student, King’s College London. Samah.alageel@kcl.ac.uk  Same as chief investigator  CV has been previously submitted to ISAC  **CV number:** 350_16  A new CV is being submitted with this protocol  An updated CV is being submitted with this protocol | | |
| 1. **List of all investigators/collaborators** (*please list the full names, affiliations and e-mail addresses* of all collaborators*, *other than the Chief Investigator*)   Other investigator:  CV has been previously submitted to ISAC  **CV number:**  A new CV is being submitted with this protocol  An updated CV is being submitted with this protocol  Other investigator:  CV has been previously submitted to ISAC  **CV number:**  A new CV is being submitted with this protocol  An updated CV is being submitted with this protocol  Other investigator:  CV has been previously submitted to ISAC  **CV number:**  A new CV is being submitted with this protocol  An updated CV is being submitted with this protocol  Other investigator:  CV has been previously submitted to ISAC  **CV number:**  A new CV is being submitted with this protocol  An updated CV is being submitted with this protocol  [Please add more investigators as necessary]**Please note that your ISAC application form and protocol* ***must*** *be copied to all e-mail addresses listed above at the time of submission of your application to the ISAC mailbox. Failure to do so will result in delays in the processing of your application.* | | |
| 1. **Conflict of interest statement*** (please provide a draft of the conflict (or competing) of interest (COI) statement that you intend to include in any publication which might result from this work)   The authors have no conflict of interest with the contents of this manuscript.  **Please refer to the International Committee of Medical Journal Editors (ICMJE) for guidance on what constitutes a COI* | | |
| 1. **Experience/expertise available** (please complete the following questions to indicate the experience/expertise available within the team of investigators/collaborators actively involved in the proposed research, including the analysis of data and interpretation of results   **Previous GPRD/CPRD Studies** **Publications using GPRD/CPRD data**  None  1-3  > 3 | | |
|  | **Yes** | **No** |
| **Is statistical expertise available within the research team?**  *If yes, please indicate the name(s) of the relevant investigator(s)*  Prof. Martin Gulliford has experience of CPRD research and relevant statistical analysis. |  |  |
| **Is experience of handling large data sets (>1 million records) available within the research team?**  *If yes, please indicate the name(s) of the relevant investigator(s)*  Prof. Martin Gulliford has several years’ experience of CPRD data analysis. |  |  |
| **Is experience of practising in UK primary care available within the research team?**  *No but we have ready access to advice from GPs within our Department.* |  |  |
| 1. **References relating to your study**   Please list up to 3 references (most relevant) relating to your proposed study:   1. Forster AS, Burgess C, Dodhia H, Fuller F, Miller J, McDermott L, et al. Do health checks improve risk factor detection in primary care? Matched cohort study using electronic health records. Journal of Public Health. 2015:fdv119. 2. Cochrane T, Davey R, Iqbal Z, Gidlow C, Kumar J, Chambers R, et al. NHS health checks through general practice: randomised trial of population cardiovascular risk reduction. BMC public health. 2012;12(1):944. 3. Artac M, Dalton AR, Majeed A, Car J, Millett C. Effectiveness of a national cardiovascular disease risk assessment program (NHS Health Check): results after one year. Preventive medicine. 2013;57(2):129-34. | | |
| **Section C: Access to the data** | | |
| 1. **Financial Sponsor of study**   Pharmaceutical Industry  *Please specify:*      Academia  *Please specify:*  Government / NHS  *Please specify:*      Charity *Please specify:*  Other  *Please specify:*Ms Alageel is supported by the Saudi Arabia Ministry of Health None | | |
| 1. **Type of Institution carrying out the analyses**   Pharmaceutical Industry *Please specify:*      Academia *Please specify:* King’s College London  Government Department *Please specify:*      Research Service Provider *Please specify:*  NHS *Please specify:*      Other  *Please specify:* | | |
| 1. **Data source**   The sponsor has direct access to CPRD GOLD and will extract the relevant data*    A data set will be supplied by CPRD**  CPRD has been commissioned to extract the relevant data and to perform the analyses  Other *Please specify:*    *If data sources other than CPRD GOLD are required, these will be supplied by CPRD  ** Please note that datasets provided by CPRD are limited in size. Applicants should contact CPRD ([KC@CPRD.com](mailto:KC@CPRD.com)) if a dataset of >300,000 patients is required. | | |
| 1. **Primary care data** (please specify which primary care data set(s) are required)   Vision only (Default for CPRD studies)  EMIS® only*  Both Vision and EMIS®*  *Note: Vision and EMIS are different clinical systems, Vision data has traditionally been used for CPRD, EMIS is currently undergoing beta-testing.*  **Investigators requiring the use of EMIS data must discuss the study with a member of CPRD staff before submitting an ISAC application*  Please list below the name of the person/s at the CPRD with whom you have discussed your request for EMIS data: | | |
| **Section D: Data linkage** | | |
| 1. **Does this protocol also seek access to data held under the CPRD Data Linkage Scheme?**   Yes*  No  If No, please move to section E.  **Investigators requiring linked data must discuss the study with a member of CPRD staff. It is important to be aware that linked data are not available for all patients in CPRD GOLD, the coverage periods for each data source may differ and charges may be applied. Please contact the CPRD Research Team on +44 (20) 3080 6383 or email* [*kc@cprd.com*](mailto:kc@cprd.com) *to discuss your requirements before submitting your application.*  Please list below the name of the person/s at the CPRD with whom you have discussed your request:  Rachael Williams, Research Statistician, CPRD  *Please note that as part of the ISAC review of linkages, the protocol may be shared - in confidence - with a representative of the requested linked data set(s) and summary details may be shared - in confidence - with the Confidentiality Advisory Group of the Health Research Authority.* | | |
| 1. **Please select the source(s) of linked data being requested:**   ONS Mortality Data  NCDR Cancer Registry Data*  Inpatient Hospital Episode Statistics  MINAP  Outpatient Hospital Episode Statistics  Mother Baby Link    Index of Multiple Deprivation  Townsend Score  Other** *Please specify:*  **Please note that applicants seeking access to cancer registry data must provide consent for publication of their study title and study institution on the UK Cancer Registry website. They must also complete a* ***Cancer Dataset Agreement Form*** *(available from CPRD) and provide a* ***System level Security Policy*** *for each organisation involved in the study.*  *** If “Other” is specified, please name an individual in CPRD that this linage has been discussed with.* | | |
| 1. **Total number of linked datasets requested including CPRD GOLD**: 3 | | |
| 1. **Is linkage to a local dataset with <1 million patients being requested?**   Yes*  No  ** If yes, please provide further details:* | | |
| 1. **If you have requested linked data sets, please indicate whether the Chief Investigator or any of the collaborators listed in response to question 5 above, have access to any of the linked datasets in a patient identifiable form, or associated with a patient index.**   Yes*  No  ** If yes, please provide further details:* | | |
| 1. **Does this study involve linking to patient *identifiable* data from other sources?**   Yes  No | | |
| **Section E: Validation/verification** | | |
| 1. **Does this protocol describe a purely observational study using CPRD data (this may include the review of anonymised free text)?**   Yes*  No**  ** Yes: If you will be using data obtained from the CPRD Group, this study does not require separate ethics approval from an NHS Research Ethics Committee.*  *** No: You may need to seek separate ethics approval from an NHS Research Ethics Committee for this study. The ISAC will provide advice on whether this may be needed.* | | |
| 1. **Does this study require anonymised free text?**   Yes*  No  **Please note that work involving free text can only be performed on the July 2013 CPRD GOLD database build or earlier versions. CPRD can provide further advice on the use of anonymised free text.* | | |
| 1. **Does this protocol involve requesting any additional information from GPs?**   Yes*  No  * *Please indicate what will be required:*  Completion of questionnaires by the GP*^ψ^* Yes  No  Provision of anonymised records (e.g. hospital discharge summaries) Yes  No  Other (please describe)  *^ψ^ Any questionnaire for completion by GPs or other health care professional must be approved by ISAC before circulation for completion.* | | |
| 1. **Does this study require contact with patients in order for them to complete a questionnaire?**   Yes*  No  **Please note that any questionnaire for completion by patients must be approved by ISAC before circulation for completion.* | | |
| 1. **Does this study require contact with patients in order to collect a sample?**   Yes*  No  ** Please state what will be collected:* | | |
| **Section F: Signatures** | | |
| 1. **Signature from the Chief Investigator**   I confirm that the above information is to the best of my knowledge accurate, and I have read and understood the guidance to applicants.  Name: M Gulliford Date: 14 April 2016 E. signature (type name): M Gulliford | | |

**Long-term outcomes of NHS Health Checks in England.**

1. **Lay Summary (Max. 200 words)**

The NHS Health Check (NHSHC) is a primary prevention programme aimed at reducing important causes of mortality in England. The programme targets individuals aged 40 to 74 years and estimates their risk of heart disease, stroke, type 2 diabetes, chronic kidney diseases and some forms of dementia. After identifying individuals at elevated risk, a personalised risk management intervention is provided. As part on ongoing evaluation of the programme’s outcomes, we aim to use CPRD data to evaluate the long-term impact of the NHSHC programme, since its introduction in 2009. In this study, we aim to evaluate changes in risk factors, risk factor management, occurrence of strokes and heart attacks and mortality outcomes. This will be done by comparing these measures between a sample who participated in the health check programme and a matched control who did not initially receive a health check. The results will provide a non-randomised assessment of the programme’s effectiveness through continuing monitoring and evaluation.

1. **Technical Summary (Max. 200 words)**

A new programme of NHS Health Checks (NHSHC) was introduced in the UK in 2009. This aims to provide cardiovascular risk assessment every five years for adults aged 40-74 who are not already diagnosed with CVD or diabetes. The NHSHC offers a promising strategy for preventing CVD, yet several concerns have been raised in relation to its uptake, cost-effectiveness, success in reducing diseases and effectiveness of risk management. In this context, evaluation of the programme’s longer-term outcomes is crucial. This study aims at evaluating changes in risk and cardiovascular clinical events and mortality outcomes following uptake of a health check. We will use a matched cohort design in the CPRD by comparing participants with a record of a complete NHSHC between 1^st^ of April 2010 and 31^st^ of March 2013 with follow-up data available for a minimum of three years after their health check up to 31^st^ March 2016, together with matched control participants who did not receive a health check. We will adopt a time-to-event framework with date of check as the start date to evaluate the onset of new prescriptions. Hazard ratios will be estimated using the cox proportional hazards model and incident rate ratios using Poisson regression to examine the association between the programme and changes in clinical events and mortality.

1. **Objectives, Specific Aims and Rationale**

**Aim:** To measure the long term outcomes of the NHS Health Checks programme and evaluate whether outcomes following an NHS Health Check are more favourable than in controls.

**Objectives:**

1. To test the hypotheses that greater changes in CVD risk factors, CVD risk score and CVD risk management follow the NHS Health Check than are observed in control participants;
2. To test the hypothesis that all-cause mortality and the incidence of clinical events (new diagnoses of ischaemic heart disease, stroke, diabetes mellitus and chronic kidney disease) may be lower in participants who received NHS Health Checks than control participants;
3. To test the hypothesis that inequalities in the outcomes of Health Checks across deprivation category may be lower in NHS Health Check participants than in control participants.
4. **Background**

Cardiovascular disease is the leading cause of death worldwide and in the United Kingdom (1). Almost 155,000 deaths annually are caused by CVD in UK, accounting for more than a quarter of all deaths (2). Diabetes also is increasing in frequency, with four million people in UK living with diabetes, which is associated with serious complications leading to disability and premature mortality (3). The NHS Health Check (NHSHC) is a primary prevention programme that was introduced in England. The programme targets individuals aged 40 to 74 years, who have not yet developed CVD and are not treated for elevated risk, and estimates their risk of heart disease, stroke, type 2 diabetes, chronic kidney diseases and some forms of dementia (4). After identifying individuals at elevated risk, a personalised risk management intervention is provided. This programme provides an opportunity in terms of addressing multiple risk factors which, if successful, might reduce the risk of several diseases. Results from the Department of Health economic model suggested that the NHS Health Check could save 650 lives, and 1600 heart attacks and 4000 cases of diabetes could be prevented a year (5).

Since the implementation of the NHS Health Check, studies have examined the effectiveness of the programme in terms of CVD risk reduction. Cochrane, Davey (6) monitored the changes in CVD risk factors after the first year of implementing the NHSHC and assessed the benefit of the lifestyle support component of the programme. They compared participants who received the health check alone with participants who received the health check together with additional lifestyle change support. The mean CVD risk declined in both groups. In the health check group, it was 32.9% at baseline and it reduced to 29.4% (p<0.001) after one year follow up. In the health check plus lifestyle support group CVD risk was 31.9% at baseline and it reduced to 29.2% (p<0.001) after one year follow up (6). Another study aimed at assessing the CVD risk reduction after one year follow-up (7). In participants who had a completed health check (n=1886) the mean global CVD risk score was reduced significantly from 28.2% (CI= 27.3,29.1) to 26.2% (CI= 25.4, 27.1)(7). Both studies showed modest reduction in CVD risk and CVD risk factors. However, participants were only followed after one year. Since the NHSHC was introduced in 2009, long term follow up would provide further assessment of the programme’s effectiveness, which this study aims to achieve.

The present analysis will draw upon our previous CPRD study on health checks (Protocol: 13_071A). (8) The previous study constructed a matched cohort design in which participants who had received health checks between 1 April 2010 and 31 March 2013 and who had at least 12 months records, were compared with controls who did not receive a health check. This previous study focused on risk factor detection and showed that health checks are associated with increased detection of hypercholesterolaemia, and to a lesser extent obesity and hypertension, but smokers may be under-represented among participants in the programme. The proposed study aims to evaluate longer term follow-up of substantive health outcomes. This study aims, therefore, to evaluate changes in risk factors, clinical events and mortality outcomes. In this proposed study, all individuals will have the potential to provide a minimum of three years of follow-up following the health check.

1. **Study Type**

The study will be hypothesis testing, estimating health check programme through a comparison of health check and control participants.

1. **Study Design**

Matched cohort study in the Clinical Practice Research Datalink (CPRD), including participants who received the NHSHC with matched control participants. This design will enable a comparison of health outcomes between participants who had the health check with control participants who attend the same general practice but did not attend the NHSHC programme.

1. **Sample Size**

Based on Forster et al. study (8) (ISAC protocol 13-071A), we intend that this study will include the same 75,123 participants with a record of a complete NHSHC between 1^st^ of April 2010 and 31^st^ of March 2013 with follow-up data available three years after their health check (up to 31/3/2016), and the same 182,245 matched control participants who did not receive the check. All individuals were eligible for data linkage as discussed in section H.

**Sample power calculation:**

Assuming that the background incidence of CHD is about 3 per 1,000 per year and the type 1 error rate is set at 0.05, if there are 75,000 health check participants and 182,000 control participants, there will be more than 90% power to detect a risk ratio of 0.85, for reduction of CHD incidence, over three years of follow-up.

1. **Data Linkage Required (if applicable)**

We have requested linkage to the Index of Multiple Deprivation data to test whether the outcomes of the NHS Health Checks differ across different socioeconomic status. We already have deprivation data obtained for protocol 13_071A but we will request all linked data again from the latest linkage Set (i.e. Set 12). This will ensure that we are working with the most accurate and consistent data (e.g. if a patient's postcode has been added or changed).

In addition, we are requesting ONS mortality data to enable cause of death analysis. We also plan to use integrated HES data to evaluate hospital admissions with ischaemic heart disease or stroke events. We are aware that a small number of patients may be eligible for linkage to IMD data but not eligible for linkage to the ONS mortality and inpatient HES data. This is because eligibility for linkage to IMD depends on the availability of a valid postcode, whereas eligibility for linkage to ONS and HES data depends on availability of a valid NHS number.

We will initially conduct the analysis using CPRD data only (the ‘unlinked analysis’). Subsequently, we will conduct a similar analysis using CPRD data together with linked data (the ‘linked analysis’). We will take into account the coverage period for the ONS mortality and inpatient HES data. Set 12 linkages provide ONS mortality and inpatient HES data through to 30/09/15, so the time period for the linked analysis will be restricted in comparison to the unlinked analysis. Patients who are not eligible for ONS and HES linkage will be excluded from the linked data analysis by restricting the study population to those eligible for all linkages. The loss of participants should not be large because the initial sample was drawn from linkage eligible participants.

1. **Study Population**

The original sample was extracted from the October 2014 release of CPRD for 140,356 participants with a record of an NHS Health Check between 1 April 2010 and 31 March 2013, from 452 general practices in England. We restricted the sample to all 106,784 participants registered with 334 general practices with IMD 2010 quintile for England, linked via the participant postcode, at the lower super-output area level. We also excluded all patients who were ever treated with antihypertensive drugs (8531) or statins (1529) before the date of the check. We also excluded patients (4859) who did not have a full year of up to standard record before the date of the check and one patient whose check was after 31 March 2013. We also excluded patients with diabetes (227), coronary heart disease (2) or stroke (3) diagnosed before the check. There remained 91,618 participants who had a health check between 1 April 2010 and 31 March 2013, who had at least 12 months record, were never treated with antihypertensive drugs or statins, and were not diagnosed with diabetes, stroke or CHD before the check. For these 91,618 participants, eligible control participants were identified for 75,123 (82%) and these participants, and their matched controls, will be included in the present study.

1. **Selection of comparison group(s) or controls**

Control participants were selected if they were eligible for the NHS Health Checks but did not receive the check, they might have been invited to attend the Health Check, or not. The control participants were individually matched by general practice, gender and age with participants who received the Health Check.

1. **Exposures, Outcomes and Covariates**

Primary outcomes: Data will be analysed for changes in all-cause mortality and CHD and stroke clinical events, diabetes diagnosis and chronic kidney disease (CKD) diagnosis. The Read codes used to identify cases of CHD in CPRD data will be those listed in reference 9. The Read codes used to identify cases of stroke in CPRD data will be those listed in reference 10. (9, 10) , while ICD-10 codes will be used in HES data. Where feasible stroke will be classified as haemorrhagic or ischaemic. CHD events will be classified as angina, myocardial infarction, coronary artery bypass surgery or percutaneous coronary intervention and other. To identify cases of diabetes, diabetes drug prescriptions will be analysed by mapping drug codes to chapter subheadings in the British National Formulary (6.1 for drugs used in diabetes). Medical diagnosis codes and levels of HbA1c levels (6.5%) will be analysed as well to identify diabetes diagnosis. Medical diagnosis codes and levels of serum creatinine will be used to identify CKD cases. (11) . Details of proposed case definitions are given in Table 1.

**Table 1: Case definitions for key measures:**

| **Condition** | **Read Codes** | **Therapy Codes** | **Test results** | **ICD-10 codes** |
| --- | --- | --- | --- | --- |
|  |  |  |  |  |
| **Ischaemic heart disease** | As listed in reference 9. | N/A | N/A | I20-I25 |
|  |  |  |  |  |
| **Stroke** | As listed in reference 10. | N/A | N/A | I60-I64 |
|  |  |  |  |  |
| **Diabetes mellitus** | As listed in Appendix 1. | BNF chapter 6.1.1 and 6.1.2 | HbA1c >=6.5% (48 mmol/mol) | E10-E14 |
|  |  |  |  |  |
| **Chronic kidney disease** | K05 and K06 codes | N/A | eGFR based on serum creatinine using CKD-EPI equation (11) | N18, N19 |
|  |  |  |  |  |

Secondary outcomes: CVD risk and CVD risk factors, including: smoking, BP, total cholesterol, HDL and BMI. Data on medication prescription for antihypertensive drugs, lipid-lowering drugs, antiplatelet drugs and nicotine replacement therapy will be analysed by recording drug codes to chapter subheadings in the British National Formulary (chapter 2 for CVD drugs).

1. **Data/ Statistical analysis**

The proportion of participants with values recorded for five risk factors (blood pressure, total cholesterol, HDL, BMI and smoking status) will be evaluated. Risk factor values will also be assessed. Changes over time in prescription of drugs for CVD risk management, including antihypertensive, lipid-lowering, antiplatelet and nicotine replacement therapy, will be evaluated.

The association between NHSHC and changes in CHD, stroke, diabetes and CKD clinical events will be examined using Cox hazard and Poisson regression models. Person-time approach will be adopted, where the start date is the date of the check. Hazard ratios will be estimated using Cox proportional hazards models and incident rate ratio using Poisson regression model. The proportional hazard assumptions will be tested. Linear mixed models will be used to study changes in continues outcomes (BP, cholesterol and BMI measurements). Follow-up will be censored if a control participant receives a health check at a later date. The effect of deprivation will be evaluated. Potential effect modification from deprivation category will be evaluated by testing for an interaction of health check status and deprivation category.

1. **Plan for addressing confounding**

To control for potential cofounders, model covariates will include age, gender, ethnicity and deprivation indices.

1. **Plan for addressing missing data**

Recording of risk factor values is an outcome of importance in respect of evaluating NHS health checks. Prescriptions, clinical events and deaths that are not recorded will be presumed to be absent, because recording of these measures is expected to be complete.

1. **Limitations of the study design, data sources and analytical methods**

A randomised evaluation would provide the optimal study design but as health checks are part of a national policy, the present non-randomised design will offer an achievable study design. We expect a high level of completeness for recording of NHS health checks because use of mandated Read codes is used for reimbursement of practices. We acknowledge that there may be lack of standardisation of blood pressure measurements and recording of measures such as smoking and body mass index may be subject to misclassification. Use of diagnostic codes for CVD events varies between general practices and some clinical events are recorded with codes that lack precision.

1. **Patient or user group involvement (if applicable)**

Patients have not contributed to the development of this protocol but we have engaged with patients in our health check evaluation work and will be able to discuss the results of this study with patient groups.

1. **Plans for disseminating and communicating study results, including the presence or absence of any restrictions on the extent and timing of publication**

The results of this study will be reported in national public health events and we will also prepare peer review paper for publication. It will also contribute to a PhD thesis.

1. **References**

| Medcode | ReadCode | ReadTerm | Type |
| --- | --- | --- | --- |
| 506 | C100112 | Non-insulin dependent diabetes mellitus | 2 |
| 608 | 66A2.00 | Follow-up diabetic assessment | 2 |
| 711 | C10..00 | Diabetes mellitus | 2 |
| 758 | C10F.00 | Type 2 diabetes mellitus | 2 |
| 1038 | C100011 | Insulin dependent diabetes mellitus | 1 |
| 1323 | F420.00 | Diabetic retinopathy | 2 |
| 1407 | C10FJ00 | Insulin treated Type 2 diabetes mellitus | 2 |
| 1549 | C10E.00 | Type 1 diabetes mellitus | 1 |
| 1647 | C108.00 | Insulin dependent diabetes mellitus | 1 |
| 1682 | C101.00 | Diabetes mellitus with ketoacidosis | 1 |
| 1684 | 66A4.00 | Diabetic on oral treatment | 2 |
| 2340 | F381311 | Diabetic amyotrophy | 2 |
| 2342 | F372.12 | Diabetic neuropathy | 2 |
| 2378 | 66AJ.00 | Diabetic - poor control | 2 |
| 2471 | K01x100 | Nephrotic syndrome in diabetes mellitus | 2 |
| 2475 | C104.11 | Diabetic nephropathy | 2 |
| 2478 | 66AJ100 | Brittle diabetes | 1 |
| 2986 | F420200 | Preproliferative diabetic retinopathy | 2 |
| 3286 | F420100 | Proliferative diabetic retinopathy | 2 |
| 3550 | 66A..00 | Diabetic monitoring | 2 |
| 3837 | F420400 | Diabetic maculopathy | 2 |
| 4513 | C109.00 | Non-insulin dependent diabetes mellitus | 2 |
| 5002 | F372.11 | Diabetic polyneuropathy | 2 |
| 5884 | C109.11 | NIDDM - Non-insulin dependent diabetes mellitus | 2 |
| 6125 | 66AS.00 | Diabetic annual review | 2 |
| 6509 | C108700 | Insulin dependent diabetes mellitus with retinopathy | 1 |
| 6791 | C108800 | Insulin dependent diabetes mellitus - poor control | 1 |
| 7045 | 14F4.00 | H/O: Admission in last year for diabetes foot problem | 2 |
| 7059 | 8H2J.00 | Admit diabetic emergency | 2 |
| 7069 | F420000 | Background diabetic retinopathy | 2 |
| 7328 | M037200 | Cellulitis in diabetic foot | 2 |
| 7563 | 66A3.00 | Diabetic on diet only | 2 |
| 7795 | C106.12 | Diabetes mellitus with neuropathy | 2 |
| 8403 | C109700 | Non-insulin dependent diabetes mellitus - poor control | 2 |
| 8414 | 8CA4100 | Pt advised re diabetic diet | 2 |
| 8836 | 66AR.00 | Diabetes management plan given | 2 |
| 8842 | 66A5.00 | Diabetic on insulin | 2 |
| 9013 | 66AJ.11 | Unstable diabetes | 2 |
| 9835 | 2BBL.00 | O/E - diabetic maculopathy present both eyes | 2 |
| 9881 | M271200 | Mixed diabetic ulcer - foot | 2 |
| 9897 | 9OL..00 | Diabetes monitoring admin. | 2 |
| 9958 | 42W..00 | Hb. A1C - diabetic control | 2 |
| 10098 | C10yy00 | Other specified diabetes mellitus with other spec comps | 2 |
| 10099 | F420300 | Advanced diabetic maculopathy | 2 |
| 10418 | C10ED00 | Type 1 diabetes mellitus with nephropathy | 1 |
| 10642 | ZC2C800 | Dietary advice for diabetes mellitus | 2 |
| 10659 | F464000 | Diabetic cataract | 2 |
| 10692 | C10EM00 | Type 1 diabetes mellitus with ketoacidosis | 1 |
| 10755 | F420600 | Non proliferative diabetic retinopathy | 2 |
| 10977 | 66Ac.00 | Diabetic peripheral neuropathy screening | 2 |
| 11018 | 8HBG.00 | Diabetic retinopathy 12 month review | 2 |
| 11047 | 66AH000 | Conversion to insulin | 2 |
| 11094 | 9NND.00 | Under care of diabetic foot screener | 2 |
| 11129 | 2BBQ.00 | O/E - left eye background diabetic retinopathy | 2 |
| 11433 | 2BBP.00 | O/E - right eye background diabetic retinopathy | 2 |
| 11471 | 8B3l.00 | Diabetes medication review | 2 |
| 11551 | C10B.00 | Diabetes mellitus induced by steroids | 2 |
| 11599 | 7276 | Pan retinal photocoagulation for diabetes | 2 |
| 11626 | F420z00 | Diabetic retinopathy NOS | 2 |
| 11663 | M271100 | Neuropathic diabetic ulcer - foot | 2 |
| 11930 | 9NN9.00 | Under care of diabetes specialist nurse | 2 |
| 12030 | 9OL6.00 | Diabetes monitoring 3rd letter | 2 |
| 12213 | 8BL2.00 | Patient on maximal tolerated therapy for diabetes | 2 |
| 12307 | 66AU.00 | Diabetes care by hospital only | 2 |
| 12455 | C10E.11 | Type I diabetes mellitus | 1 |
| 12506 | 66AP.00 | Diabetes: practice programme | 2 |
| 12640 | C10FC00 | Type 2 diabetes mellitus with nephropathy | 2 |
| 12675 | 66AQ.00 | Diabetes: shared care programme | 2 |
| 12682 | 679R.00 | Patient offered diabetes structured education programme | 2 |
| 12736 | C10F500 | Type 2 diabetes mellitus with gangrene | 2 |
| 13057 | 679L.00 | Health education - diabetes | 2 |
| 13067 | 66AZ.00 | Diabetic monitoring NOS | 2 |
| 13069 | 66A8.00 | Has seen dietician - diabetes | 2 |
| 13070 | 66A1.00 | Initial diabetic assessment | 2 |
| 13071 | 66AI.00 | Diabetic - good control | 2 |
| 13074 | 13B1.00 | Diabetic diet | 2 |
| 13078 | 13AC.00 | Diabetic weight reducing diet | 2 |
| 13097 | 2BBT.00 | O/E - right eye proliferative diabetic retinopathy | 2 |
| 13099 | 2BBR.00 | O/E - right eye preproliferative diabetic retinopathy | 2 |
| 13101 | 2BBV.00 | O/E - left eye proliferative diabetic retinopathy | 2 |
| 13102 | 2BBW.00 | O/E - right eye diabetic maculopathy | 2 |
| 13103 | 2BBS.00 | O/E - left eye preproliferative diabetic retinopathy | 2 |
| 13108 | 2BBX.00 | O/E - left eye diabetic maculopathy | 2 |
| 13191 | 9OL..11 | Diabetes clinic administration | 2 |
| 13192 | 9OLA.00 | Diabetes monitor. check done | 2 |
| 13194 | 9OL4.00 | Diabetes monitoring 1st letter | 2 |
| 13195 | 9OL5.00 | Diabetes monitoring 2nd letter | 2 |
| 13196 | 66AD.00 | Fundoscopy - diabetic check | 2 |
| 13197 | 9OL1.00 | Attends diabetes monitoring | 2 |
| 13279 | C104y00 | Other specified diabetes mellitus with renal complications | 2 |
| 14049 | 42WZ.00 | Hb. A1C - diabetic control NOS | 2 |
| 14050 | 42c..00 | HbA1 - diabetic control | 2 |
| 14560 | 4493 | Serum insulin | 2 |
| 14803 | C100100 | Diabetes mellitus, adult onset, no mention of complication | 2 |
| 14889 | C100111 | Maturity onset diabetes | 2 |
| 15690 | C103.00 | Diabetes mellitus with ketoacidotic coma | 1 |
| 16230 | C106.00 | Diabetes mellitus with neurological manifestation | 2 |
| 16490 | 66AH.00 | Diabetic treatment changed | 2 |
| 16491 | C106.13 | Diabetes mellitus with polyneuropathy | 2 |
| 16502 | C104.00 | Diabetes mellitus with renal manifestation | 2 |
| 16881 | ZV65312 | [V]Dietary counselling in diabetes mellitus | 2 |
| 17067 | F171100 | Autonomic neuropathy due to diabetes | 2 |
| 17095 | 2G5A.00 | O/E - Right diabetic foot at risk | 2 |
| 17247 | F35z000 | Diabetic mononeuritis NOS | 2 |
| 17262 | C109600 | Non-insulin-dependent diabetes mellitus with retinopathy | 2 |
| 17313 | F440700 | Diabetic iritis | 2 |
| 17545 | C108F11 | Type I diabetes mellitus with diabetic cataract | 1 |
| 17817 | 7L19800 | Subcutaneous injection of insulin | 2 |
| 17858 | C108.12 | Type 1 diabetes mellitus | 1 |
| 17859 | C109.12 | Type 2 diabetes mellitus | 2 |
| 17869 | 66AL.00 | Diabetic-uncooperative patient | 2 |
| 17886 | 66AM.00 | Diabetic - follow-up default | 2 |
| 18056 | 2G5C.00 | Foot abnormality - diabetes related | 2 |
| 18066 | 8CE0.00 | Diabetic leaflet given | 2 |
| 18142 | N030000 | Diabetic cheiroarthropathy | 2 |
| 18143 | C109G11 | Type II diabetes mellitus with arthropathy | 2 |
| 18167 | 66AT.00 | Annual diabetic blood test | 2 |
| 18209 | C109012 | Type 2 diabetes mellitus with renal complications | 2 |
| 18219 | C109.13 | Type II diabetes mellitus | 2 |
| 18230 | C108J12 | Type 1 diabetes mellitus with neuropathic arthropathy | 1 |
| 18264 | C109J12 | Insulin treated Type II diabetes mellitus | 2 |
| 18278 | C109J00 | Insulin treated Type 2 diabetes mellitus | 2 |
| 18311 | 68A7.00 | Diabetic retinopathy screening | 2 |
| 18387 | C10E700 | Type 1 diabetes mellitus with retinopathy | 1 |
| 18390 | C10FM00 | Type 2 diabetes mellitus with persistent microalbuminuria | 2 |
| 18425 | C10FB00 | Type 2 diabetes mellitus with polyneuropathy | 2 |
| 18496 | C10F600 | Type 2 diabetes mellitus with retinopathy | 2 |
| 18505 | C108.11 | IDDM-Insulin dependent diabetes mellitus | 1 |
| 18642 | C10EH00 | Type 1 diabetes mellitus with arthropathy | 1 |
| 18662 | 8HBH.00 | Diabetic retinopathy 6 month review | 2 |
| 18683 | C10E500 | Type 1 diabetes mellitus with ulcer | 1 |
| 18777 | C10F000 | Type 2 diabetes mellitus with renal complications | 2 |
| 19739 | 68A9.00 | Diabetic retinopathy screening offered | 2 |
| 20696 | 66AA.11 | Injection sites - diabetic | 2 |
| 20900 | 9OLA.11 | Diabetes monitored | 2 |
| 21420 | 66AJ200 | Loss of hypoglycaemic warning |  |
| 21482 | C102.00 | Diabetes mellitus with hyperosmolar coma | 2 |
| 21689 | 13AB.00 | Diabetic lipid lowering diet | 2 |
| 21983 | C108012 | Type 1 diabetes mellitus with renal complications | 1 |
| 22023 | 66AJz00 | Diabetic - poor control NOS | 2 |
| 22130 | 9OL3.00 | Diabetes monitoring default | 2 |
| 22487 | C10N.00 | Secondary diabetes mellitus | 2 |
| 22573 | C106z00 | Diabetes mellitus NOS with neurological manifestation | 2 |
| 22823 | 66Ab.00 | Diabetic foot examination | 2 |
| 22871 | C10EP00 | Type 1 diabetes mellitus with exudative maculopathy | 1 |
| 22884 | C10F.11 | Type II diabetes mellitus | 2 |
| 22967 | 2BBF.00 | Retinal abnormality - diabetes related | 2 |
| 23340 | SL23400 | Insulin poisoning | 2 |
| 23479 | C350011 | Bronzed diabetes | 2 |
| 24327 | M271000 | Ischaemic ulcer diabetic foot | 2 |
| 24363 | 8A13.00 | Diabetic stabilisation | 2 |
| 24423 | C108.13 | Type I diabetes mellitus | 1 |
| 24458 | C109711 | Type II diabetes mellitus - poor control | 2 |
| 24490 | C100000 | Diabetes mellitus, juvenile type, no mention of complication | 1 |
| 24571 | F372200 | Asymptomatic diabetic neuropathy | 2 |
| 24693 | C109G00 | Non-insulin dependent diabetes mellitus with arthropathy | 2 |
| 24694 | C108B00 | Insulin dependent diabetes mellitus with mononeuropathy | 1 |
| 24836 | C109C12 | Type 2 diabetes mellitus with nephropathy | 2 |
| 25041 | ZC2CA00 | Dietary advice for type II diabetes | 2 |
| 25591 | C10FQ00 | Type 2 diabetes mellitus with exudative maculopathy | 2 |
| 25627 | C10F700 | Type 2 diabetes mellitus - poor control | 2 |
| 25636 | 66Aa.00 | Diabetic diet - poor compliance | 2 |
| 26054 | C10FL00 | Type 2 diabetes mellitus with persistent proteinuria | 2 |
| 26108 | C10B000 | Steroid induced diabetes mellitus without complication | 2 |
| 26603 | 9OL2.00 | Refuses diabetes monitoring | 2 |
| 26604 | 66AY.00 | Diabetic diet - good compliance | 2 |
| 26605 | 9OLB.00 | Attended diabetes structured education programme | 2 |
| 26664 | 2G5B.00 | O/E - Left diabetic foot at risk | 2 |
| 26666 | 2G5E.00 | O/E - Right diabetic foot at low risk | 2 |
| 26667 | 2G5I.00 | O/E - Left diabetic foot at low risk | 2 |
| 26855 | C108400 | Unstable insulin dependent diabetes mellitus | 1 |
| 27891 | N030100 | Diabetic Charcot arthropathy | 2 |
| 27921 | 2G51000 | Foot abnormality - diabetes related | 2 |
| 28769 | 66AV.00 | Diabetic on insulin and oral treatment | 2 |
| 28856 | 8CP2.00 | Transition of diabetes care options discussed | 2 |
| 28873 | 66Ai.00 | Diabetic 6 month review | 2 |
| 29041 | 66AN.00 | Date diabetic treatment start | 2 |
| 29979 | C109900 | Non-insulin-dependent diabetes mellitus without complication | 2 |
| 30247 | TJ23000 | Adverse reaction to insulins | 2 |
| 30294 | C10EL00 | Type 1 diabetes mellitus with persistent microalbuminuria | 1 |
| 30323 | C10EK00 | Type 1 diabetes mellitus with persistent proteinuria | 1 |
| 30477 | F420700 | High risk proliferative diabetic retinopathy | 2 |
| 31053 | R054300 | [D]Widespread diabetic foot gangrene | 2 |
| 31141 | 9OL8.00 | Diabetes monitor.phone invite | 2 |
| 31156 | 2G5J.00 | O/E - Left diabetic foot at moderate risk | 2 |
| 31157 | 2G5F.00 | O/E - Right diabetic foot at moderate risk | 2 |
| 31171 | 2G5G.00 | O/E - Right diabetic foot at high risk | 2 |
| 31172 | 2G5K.00 | O/E - Left diabetic foot at high risk | 2 |
| 31240 | 9OL7.00 | Diabetes monitor.verbal invite | 2 |
| 31241 | 9OLZ.00 | Diabetes monitoring admin.NOS | 2 |
| 31310 | C108900 | Insulin dependent diabetes maturity onset | 1 |
| 31529 | U602300 | [X]Insul/oral hypoglyc drugs caus adverse eff therapeut use | 2 |
| 31790 | F372.00 | Polyneuropathy in diabetes | 2 |
| 32193 | C11y000 | Steroid induced diabetes | 2 |
| 32359 | ZRbH.00 | Perceived control of insulin-dependent diabetes | 1 |
| 32403 | C107.11 | Diabetes mellitus with gangrene | 2 |
| 32556 | C107.12 | Diabetes with gangrene | 2 |
| 32619 | 66Af.00 | Patient diabetes education review | 2 |
| 32627 | C10FN00 | Type 2 diabetes mellitus with ketoacidosis | 2 |
| 32770 | 44V3.00 | Glucose tol. test diabetic | 2 |
| 33254 | C105.00 | Diabetes mellitus with ophthalmic manifestation | 2 |
| 33343 | C10y.00 | Diabetes mellitus with other specified manifestation | 2 |
| 33807 | C107200 | Diabetes mellitus, adult with gangrene | 2 |
| 33969 | C10A100 | Malnutrition-related diabetes mellitus with ketoacidosis | 2 |
| 34152 | G73y000 | Diabetic peripheral angiopathy | 2 |
| 34268 | C10F200 | Type 2 diabetes mellitus with neurological complications | 2 |
| 34283 | C105z00 | Diabetes mellitus NOS with ophthalmic manifestation | 2 |
| 34450 | C10FK00 | Hyperosmolar non-ketotic state in type 2 diabetes mellitus | 2 |
| 34528 | 3882 | Diabetes well being questionnaire | 2 |
| 34912 | C109400 | Non-insulin dependent diabetes mellitus with ulcer | 2 |
| 35105 | C104100 | Diabetes mellitus, adult onset, with renal manifestation | 2 |
| 35107 | C104z00 | Diabetes mellitus with nephropathy NOS | 2 |
| 35116 | 2G5L.00 | O/E - Left diabetic foot - ulcerated | 2 |
| 35288 | C10E800 | Type 1 diabetes mellitus - poor control | 1 |
| 35316 | 2G5H.00 | O/E - Right diabetic foot - ulcerated | 2 |
| 35321 | 8H3O.00 | Non-urgent diabetic admission | 2 |
| 35385 | C10FH00 | Type 2 diabetes mellitus with neuropathic arthropathy | 2 |
| 35399 | C107.00 | Diabetes mellitus with peripheral circulatory disorder | 2 |
| 35785 | F372100 | Chronic painful diabetic neuropathy | 2 |
| 36633 | C109K00 | Hyperosmolar non-ketotic state in type 2 diabetes mellitus | 2 |
| 36695 | C10D.00 | Diabetes mellitus autosomal dominant type 2 | 2 |
| 36798 | 7L10000 | Continuous subcutaneous infusion of insulin | 2 |
| 37315 | F3y0.00 | Diabetic mononeuropathy | 2 |
| 37648 | C109J11 | Insulin treated non-insulin dependent diabetes mellitus | 2 |
| 37806 | C10FF00 | Type 2 diabetes mellitus with peripheral angiopathy | 2 |
| 38076 | M21yC00 | Insulin lipohypertrophy | 2 |
| 38078 | 66A9.00 | Understands diet - diabetes | 2 |
| 38130 | ZRB6.00 | Diabetes wellbeing questionnaire | 2 |
| 38161 | C108711 | Type I diabetes mellitus with retinopathy | 1 |
| 38617 | C101y00 | Other specified diabetes mellitus with ketoacidosis | 1 |
| 38986 | C100.00 | Diabetes mellitus with no mention of complication | 2 |
| 39070 | C10EE00 | Type 1 diabetes mellitus with hypoglycaemic coma | 1 |
| 39317 | C106100 | Diabetes mellitus, adult onset, + neurological manifestation | 2 |
| 39420 | F381300 | Myasthenic syndrome due to diabetic amyotrophy | 2 |
| 39809 | C108J00 | Insulin dependent diab mell with neuropathic arthropathy | 1 |
| 40023 | C102000 | Diabetes mellitus, juvenile type, with hyperosmolar coma | 1 |
| 40216 | 449D.00 | Plasma insulin level | 2 |
| 40401 | C109500 | Non-insulin dependent diabetes mellitus with gangrene | 2 |
| 40682 | C10E900 | Type 1 diabetes mellitus maturity onset | 1 |
| 40837 | C10EN00 | Type 1 diabetes mellitus with ketoacidotic coma | 1 |
| 40933 | 4494 | Serum insulin - C -polypeptide | 2 |
| 40962 | C109H00 | Non-insulin dependent d m with neuropathic arthropathy | 2 |
| 41049 | C108712 | Type 1 diabetes mellitus with retinopathy | 1 |
| 41389 | C105100 | Diabetes mellitus, adult onset, + ophthalmic manifestation | 2 |
| 41686 | Cyu2000 | [X]Other specified diabetes mellitus | 2 |
| 41716 | C108C00 | Insulin dependent diabetes mellitus with polyneuropathy | 1 |
| 42505 | C101z00 | Diabetes mellitus NOS with ketoacidosis | 1 |
| 42567 | C103000 | Diabetes mellitus, juvenile type, with ketoacidotic coma | 1 |
| 42729 | C108E11 | Type I diabetes mellitus with hypoglycaemic coma | 1 |
| 42762 | C109612 | Type 2 diabetes mellitus with retinopathy | 2 |
| 42831 | C10E200 | Type 1 diabetes mellitus with neurological complications | 1 |
| 43139 | C102100 | Diabetes mellitus, adult onset, with hyperosmolar coma | 2 |
| 43227 | C10F311 | Type II diabetes mellitus with multiple complications | 2 |
| 43453 | C10C.00 | Diabetes mellitus autosomal dominant | 2 |
| 43493 | M21yC11 | Insulin site lipohypertrophy | 2 |
| 43785 | C109D00 | Non-insulin dependent diabetes mellitus with hypoglyca coma | 2 |
| 43857 | C10M.00 | Lipoatrophic diabetes mellitus | 2 |
| 43921 | C10E400 | Unstable type 1 diabetes mellitus | 1 |
| 43951 | 66AK.00 | Diabetic - cooperative patient | 2 |
| 44033 | F345000 | Diabetic mononeuritis multiplex | 2 |
| 44260 | C108F00 | Insulin dependent diabetes mellitus with diabetic cataract | 1 |
| 44312 | 9M10.00 | Informed dissent for diabetes national audit | 2 |
| 44440 | C108E00 | Insulin dependent diabetes mellitus with hypoglycaemic coma | 1 |
| 44443 | C108500 | Insulin dependent diabetes mellitus with ulcer | 1 |
| 44779 | C109E12 | Type 2 diabetes mellitus with diabetic cataract | 2 |
| 44982 | C10FE00 | Type 2 diabetes mellitus with diabetic cataract | 2 |
| 45250 | ZL22500 | Under care of diabetic liaison nurse | 2 |
| 45276 | C10E312 | Insulin dependent diabetes mellitus with multiple complicat | 1 |
| 45467 | C109B00 | Non-insulin dependent diabetes mellitus with polyneuropathy | 2 |
| 45491 | C10z.00 | Diabetes mellitus with unspecified complication | 2 |
| 45913 | C109712 | Type 2 diabetes mellitus - poor control | 2 |
| 45914 | C108812 | Type 1 diabetes mellitus - poor control | 1 |
| 45919 | C109212 | Type 2 diabetes mellitus with neurological complications | 2 |
| 46150 | C109512 | Type 2 diabetes mellitus with gangrene | 2 |
| 46290 | C108y00 | Other specified diabetes mellitus with multiple comps | 2 |
| 46301 | C10EC00 | Type 1 diabetes mellitus with polyneuropathy | 1 |
| 46624 | C10C.11 | Maturity onset diabetes in youth | 2 |
| 46850 | C108811 | Type I diabetes mellitus - poor control | 1 |
| 46917 | C10FD00 | Type 2 diabetes mellitus with hypoglycaemic coma | 2 |
| 46963 | C108000 | Insulin-dependent diabetes mellitus with renal complications | 1 |
| 47011 | 8Hj0.00 | Referral to diabetes structured education programme | 2 |
| 47032 | 8CS0.00 | Diabetes care plan agreed | 2 |
| 47058 | 8Hg4.00 | Discharged from care of diabetes specialist nurse | 2 |
| 47315 | C10F711 | Type II diabetes mellitus - poor control | 2 |
| 47321 | C10F100 | Type 2 diabetes mellitus with ophthalmic complications | 2 |
| 47328 | 2BBk.00 | O/E - right eye stable treated prolif diabetic retinopathy | 2 |
| 47341 | 8A12.00 | Diabetic crisis monitoring | 2 |
| 47377 | C105y00 | Other specified diabetes mellitus with ophthalmic complicatn | 2 |
| 47409 | C109B11 | Type II diabetes mellitus with polyneuropathy | 2 |
| 47582 | C10E000 | Type 1 diabetes mellitus with renal complications | 1 |
| 47584 | F420500 | Advanced diabetic retinal disease | 2 |
| 47649 | C10E100 | Type 1 diabetes mellitus with ophthalmic complications | 1 |
| 47650 | C10E300 | Type 1 diabetes mellitus with multiple complications | 1 |
| 47816 | C109H11 | Type II diabetes mellitus with neuropathic arthropathy | 2 |
| 47954 | C10F900 | Type 2 diabetes mellitus without complication | 2 |
| 48078 | F372000 | Acute painful diabetic neuropathy | 2 |
| 48192 | C109E11 | Type II diabetes mellitus with diabetic cataract | 2 |
| 48310 | ZV6DA00 | [V]Admitted for commencement of insulin | 2 |
| 49074 | C10F400 | Type 2 diabetes mellitus with ulcer | 2 |
| 49146 | C108211 | Type I diabetes mellitus with neurological complications | 1 |
| 49276 | C108100 | Insulin-dependent diabetes mellitus with ophthalmic comps | 1 |
| 49554 | C10EF00 | Type 1 diabetes mellitus with diabetic cataract | 1 |
| 49640 | 2G5W.00 | O/E - left chronic diabetic foot ulcer | 2 |
| 49655 | C10F611 | Type II diabetes mellitus with retinopathy | 2 |
| 49869 | C109G12 | Type 2 diabetes mellitus with arthropathy | 2 |
| 49884 | 6761 | Diabetic pre-pregnancy counselling | 2 |
| 49949 | C10E411 | Unstable type I diabetes mellitus | 1 |
| 50175 | 66AW.00 | Diabetic foot risk assessment | 2 |
| 50225 | C109011 | Type II diabetes mellitus with renal complications | 2 |
| 50429 | C109100 | Non-insulin-dependent diabetes mellitus with ophthalm comps | 2 |
| 50527 | C10FB11 | Type II diabetes mellitus with polyneuropathy | 2 |
| 50813 | C109A11 | Type II diabetes mellitus with mononeuropathy | 2 |
| 50972 | C100z00 | Diabetes mellitus NOS with no mention of complication | 2 |
| 51261 | C10E.12 | Insulin dependent diabetes mellitus | 1 |
| 51697 | C10G.00 | Secondary pancreatic diabetes mellitus | 2 |
| 51756 | C10FP00 | Type 2 diabetes mellitus with ketoacidotic coma | 2 |
| 51939 | ZV6DB00 | [V]Admitted for conversion to insulin | 2 |
| 51957 | C108511 | Type I diabetes mellitus with ulcer | 1 |
| 52041 | 2BBl.00 | O/E - left eye stable treated prolif diabetic retinopathy | 2 |
| 52104 | C108300 | Insulin dependent diabetes mellitus with multiple complicatn | 1 |
| 52212 | Cyu2.00 | [X]Diabetes mellitus | 2 |
| 52236 | C10A.00 | Malnutrition-related diabetes mellitus | 2 |
| 52237 | 9360 | Patient held diabetic record issued | 2 |
| 52283 | C108200 | Insulin-dependent diabetes mellitus with neurological comps | 1 |
| 52303 | C109000 | Non-insulin-dependent diabetes mellitus with renal comps | 2 |
| 52630 | 2BBo.00 | O/E - sight threatening diabetic retinopathy | 2 |
| 53200 | C101000 | Diabetes mellitus, juvenile type, with ketoacidosis | 1 |
| 53238 | 66AG.00 | Diabetic drug side effects | 2 |
| 53303 | 449E.00 | Plasma insulin C-peptide level | 2 |
| 53392 | C10F911 | Type II diabetes mellitus without complication | 2 |
| 53634 | R054200 | [D]Gangrene of toe in diabetic | 2 |
| 54008 | C10EJ00 | Type 1 diabetes mellitus with neuropathic arthropathy | 1 |
| 54212 | C109F00 | Non-insulin-dependent d m with peripheral angiopath | 2 |
| 54600 | C10E412 | Unstable insulin dependent diabetes mellitus | 1 |
| 54601 | 9NN8.00 | Under care of diabetologist | 2 |
| 54856 | C101100 | Diabetes mellitus, adult onset, with ketoacidosis | |
| 54899 | C109F11 | Type II diabetes mellitus with peripheral angiopathy | 2 |
| 55075 | C109411 | Type II diabetes mellitus with ulcer | 2 |
| 55239 | C10EQ00 | Type 1 diabetes mellitus with gastroparesis | 1 |
| 55842 | C109200 | Non-insulin-dependent diabetes mellitus with neuro comps | 2 |
| 56268 | C109D11 | Type II diabetes mellitus with hypoglycaemic coma | 2 |
| 56448 | C108A00 | Insulin-dependent diabetes without complication | 1 |
| 56803 | C107400 | NIDDM with peripheral circulatory disorder | 2 |
| 57278 | C10F011 | Type II diabetes mellitus with renal complications | 2 |
| 57333 | N030011 | Diabetic cheiropathy | 2 |
| 57389 | 93C4.00 | Patient consent given for addition to diabetic register | 2 |
| 57621 | C108D00 | Insulin dependent diabetes mellitus with nephropathy | 1 |
| 58133 | ZLD7500 | Discharge by diabetic liaison nurse | 2 |
| 58604 | C109611 | Type II diabetes mellitus with retinopathy | 2 |
| 59253 | C10FG00 | Type 2 diabetes mellitus with arthropathy | 2 |
| 59288 | C103y00 | Other specified diabetes mellitus with coma | 2 |
| 59365 | C109C00 | Non-insulin dependent diabetes mellitus with nephropathy | 2 |
| 59725 | C109111 | Type II diabetes mellitus with ophthalmic complications | 2 |
| 59903 | C106.11 | Diabetic amyotrophy | 2 |
| 59991 | C10D.11 | Maturity onset diabetes in youth type 2 | 2 |
| 60107 | C108411 | Unstable type I diabetes mellitus | 1 |
| 60208 | C108J11 | Type I diabetes mellitus with neuropathic arthropathy | 1 |
| 60499 | C108600 | Insulin dependent diabetes mellitus with gangrene | 1 |
| 60699 | C109F12 | Type 2 diabetes mellitus with peripheral angiopathy | 2 |
| 60796 | C10FL11 | Type II diabetes mellitus with persistent proteinuria | 2 |
| 61021 | 68AB.00 | Diabetic digital retinopathy screening offered | 2 |
| 61049 | 449G.00 | Serum pro-insulin level | 2 |
| 61071 | C109D12 | Type 2 diabetes mellitus with hypoglycaemic coma | 2 |
| 61122 | C10H.00 | Diabetes mellitus induced by non-steroid drugs | 2 |
| 61210 | TJ23z00 | Adverse reaction to insulins and antidiabetic agents NOS | 2 |
| 61344 | C108011 | Type I diabetes mellitus with renal complications | 1 |
| 61461 | 9M00.00 | Informed consent for diabetes national audit | 2 |
| 61470 | 66Al.00 | Diabetic monitoring - higher risk albumin excretion | 2 |
| 61523 | C106y00 | Other specified diabetes mellitus with neurological comps | 2 |
| 61829 | C108212 | Type 1 diabetes mellitus with neurological complications | 1 |
| 62107 | C109511 | Type II diabetes mellitus with gangrene | 2 |
| 62146 | C109300 | Non-insulin-dependent diabetes mellitus with multiple comps | 2 |
| 62209 | C10EM11 | Type I diabetes mellitus with ketoacidosis | 1 |
| 62352 | C108H11 | Type I diabetes mellitus with arthropathy | 1 |
| 62384 | 2G5V.00 | O/E - right chronic diabetic foot ulcer | 2 |
| 62613 | C10EA11 | Type I diabetes mellitus without complication | 1 |
| 62674 | C10FA00 | Type 2 diabetes mellitus with mononeuropathy | 2 |
| 63017 | C108911 | Type I diabetes mellitus maturity onset | 1 |
| 63357 | C107100 | Diabetes mellitus, adult, + peripheral circulatory disorder | 2 |
| 63364 | U602312 | [X] Adverse reaction to insulins | 2 |
| 63371 | C10y100 | Diabetes mellitus, adult, + other specified manifestation | 2 |
| 63412 | 8CR2.00 | Diabetes clinical management plan | 2 |
| 63690 | C10FR00 | Type 2 diabetes mellitus with gastroparesis | 2 |
| 63762 | C10z100 | Diabetes mellitus, adult onset, + unspecified complication | 2 |
| 64283 | C10zy00 | Other specified diabetes mellitus with unspecified comps | 2 |
| 64357 | C10zz00 | Diabetes mellitus NOS with unspecified complication | 2 |
| 64446 | C108G00 | Insulin dependent diab mell with peripheral angiopathy | 1 |
| 64449 | C108z00 | Unspecified diabetes mellitus with multiple complications | 2 |
| 64571 | C109C11 | Type II diabetes mellitus with nephropathy | 2 |
| 64668 | C10FJ11 | Insulin treated Type II diabetes mellitus | 2 |
| 65025 | C107z00 | Diabetes mellitus NOS with peripheral circulatory disorder | 2 |
| 65062 | C103z00 | Diabetes mellitus NOS with ketoacidotic coma | 1 |
| 65267 | C10F300 | Type 2 diabetes mellitus with multiple complications | 2 |
| 65463 | F420800 | High risk non proliferative diabetic retinopathy | 2 |
| 65616 | C108H00 | Insulin dependent diabetes mellitus with arthropathy | 1 |
| 65684 | U602311 | [X] Adverse reaction to insulins and antidiabetic agents | 2 |
| 65704 | C109412 | Type 2 diabetes mellitus with ulcer | 2 |
| 66145 | C10EN11 | Type I diabetes mellitus with ketoacidotic coma | 1 |
| 66274 | 66Ah.00 | Insulin needles changed for each injection | 2 |
| 66475 | 66Ak.00 | Diabetic monitoring - lower risk albumin excretion | 2 |
| 66675 | C10A000 | Malnutrition-related diabetes mellitus with coma | 2 |
| 66872 | C108D11 | Type I diabetes mellitus with nephropathy | 1 |
| 66965 | C109H12 | Type 2 diabetes mellitus with neuropathic arthropathy | 2 |
| 67212 | C10H000 | DM induced by non-steroid drugs without complication | 2 |
| 67664 | ZRBa.00 | Education score - diabetes | 2 |
| 67853 | C106000 | Diabetes mellitus, juvenile, + neurological manifestation | 1 |
| 67905 | C109211 | Type II diabetes mellitus with neurological complications | 2 |
| 68105 | C10EB00 | Type 1 diabetes mellitus with mononeuropathy | 1 |
| 68390 | C108512 | Type 1 diabetes mellitus with ulcer | 1 |
| 68546 | ZRB4.00 | Diabetes clinic satisfaction questionnaire | 2 |
| 68714 | SL23.00 | Insulins and antidiabetic poisoning | 2 |
| 68792 | C10z000 | Diabetes mellitus, juvenile type, + unspecified complication | 1 |
| 68818 | ZRB5.11 | DTSQ - Diabetes treatment satisfaction questionnaire | 2 |
| 68843 | C103100 | Diabetes mellitus, adult onset, with ketoacidotic coma | 2 |
| 68928 | TJ23.00 | Adverse reaction to insulins and antidiabetic agents | 2 |
| 69043 | ZC2C900 | Dietary advice for type I diabetes | 1 |
| 69124 | C107300 | IDDM with peripheral circulatory disorder | 1 |
| 69152 | 66Aj.00 | Insulin needles changed less than once a day | 2 |
| 69278 | C109E00 | Non-insulin depend diabetes mellitus with diabetic cataract | 2 |
| 69676 | C10EA00 | Type 1 diabetes mellitus without complication | 1 |
| 69748 | C105000 | Diabetes mellitus, juvenile type, + ophthalmic manifestation | 1 |
| 69993 | C10E600 | Type 1 diabetes mellitus with gangrene | 1 |
| 70316 | C109112 | Type 2 diabetes mellitus with ophthalmic complications | 2 |
| 70448 | C107000 | Diabetes mellitus, juvenile +peripheral circulatory disorder | 1 |
| 70766 | C108E12 | Type 1 diabetes mellitus with hypoglycaemic coma | 1 |
| 70821 | C10yz00 | Diabetes mellitus NOS with other specified manifestation | 2 |
| 72320 | C109A00 | Non-insulin dependent diabetes mellitus with mononeuropathy | 2 |
| 72333 | 8HME.00 | Listed for Diabetology admissn | 2 |
| 72345 | C102z00 | Diabetes mellitus NOS with hyperosmolar coma | 2 |
| 72702 | C10E812 | Insulin dependent diabetes mellitus - poor control | 1 |
| 83485 | 66Am.00 | Insulin dose changed | 2 |
| 83532 | 66Ao.00 | Diabetes type 2 review | 2 |
| 85660 | 66An.00 | Diabetes type 1 review | 1 |
| 85991 | C10FM11 | Type II diabetes mellitus with persistent microalbuminuria | 2 |
| 90301 | 66Ag.00 | Insulin needles changed daily | 2 |
| 91164 | ZRB4.11 | CSQ - Diabetes clinic satisfaction questionnaire | 2 |
| 91646 | C10F411 | Type II diabetes mellitus with ulcer | 2 |
| 91942 | C10E311 | Type I diabetes mellitus with multiple complications | 1 |
| 91943 | C10EC11 | Type I diabetes mellitus with polyneuropathy | 1 |
| 93390 | 9OLH.00 | Attended DAFNE diabetes structured education programme | 1 |
| 93468 | C10EG00 | Type 1 diabetes mellitus with peripheral angiopathy | 1 |
| 93491 | 9OLJ.00 | DAFNE diabetes structured education programme completed | 1 |
| 93529 | 9OLK.00 | DESMOND diabetes structured education programme completed | 2 |
| 93631 | 9OLL.00 | XPERT diabetes structured education programme completed | 2 |
| 93657 | 8Hj4.00 | Referral to DESMOND diabetes structured education programme | 2 |
| 93704 | 8Hj3.00 | Referral to DAFNE diabetes structured education programme | 1 |
| 93727 | C10FE11 | Type II diabetes mellitus with diabetic cataract | 2 |
| 93854 | 9OLM.00 | Diabetes structured education programme declined | 2 |
| 93870 | 8Hj5.00 | Referral to XPERT diabetes structured education programme | 2 |
| 93875 | C10E712 | Insulin dependent diabetes mellitus with retinopathy | 1 |
| 93878 | C10E511 | Type I diabetes mellitus with ulcer | 1 |
| 93922 | C104000 | Diabetes mellitus, juvenile type, with renal manifestation | 1 |
| 94011 | 9OLG.00 | Attended XPERT diabetes structured education programme | 2 |
| 94186 | 9OLF.00 | Diabetes structured education programme completed | 2 |
| 94383 | C10N000 | Secondary diabetes mellitus without complication | 2 |
| 94699 | ZRB5.00 | Diabetes treatment satisfaction questionnaire | 2 |
| 94955 | 9NiE.00 | Did not attend XPERT diabetes structured education programme | 2 |
| 94956 | 8I84.00 | Did not complete XPERT diabetes structured education program | 2 |
| 95093 | 8I83.00 | Did not complete DESMOND diabetes structured educat program | 2 |
| 95094 | 8I81.00 | Did not complete diabetes structured education programme | 2 |
| 95159 | 9NiD.00 | Did not attend DESMOND diabetes structured education program | 2 |
| 95343 | C10E711 | Type I diabetes mellitus with retinopathy | 1 |
| 95351 | C10FA11 | Type II diabetes mellitus with mononeuropathy | 2 |
| 95553 | 9NiA.00 | Did not attend diabetes structured education programme | 2 |
| 95636 | C10ER00 | Latent autoimmune diabetes mellitus in adult | 1 |
| 95992 | C108A11 | Type I diabetes mellitus without complication | 1 |
| 95994 | 66Aq.00 | Diabetic foot screen | 2 |
| 96010 | 66Ap.00 | Insulin treatment initiated | 2 |
| 96143 | 9kL..00 | Insulin initiation - enhanced services administration | 2 |
| 96235 | C10E911 | Type I diabetes mellitus maturity onset | 1 |
| 96506 | C10G000 | Secondary pancreatic diabetes mellitus without complication | 2 |
| 97446 | C108912 | Type 1 diabetes mellitus maturity onset | 1 |
| 97474 | C108412 | Unstable type 1 diabetes mellitus | 1 |
| 97809 | 8I82.00 | Did not complete DAFNE diabetes structured education program | 1 |
| 97824 | ZRB6.11 | DWBQ - Diabetes wellbeing questionnaire | 2 |
| 97849 | C10E912 | Insulin dependent diabetes maturity onset | 1 |
| 97894 | C10EP11 | Type I diabetes mellitus with exudative maculopathy | 1 |
| 98071 | C10E112 | Insulin-dependent diabetes mellitus with ophthalmic comps | 1 |
| 98392 | C10C.12 | Maturity onset diabetes in youth type 1 | 1 |
| 98616 | C10F211 | Type II diabetes mellitus with neurological complications | 2 |
| 98704 | C10E512 | Insulin dependent diabetes mellitus with ulcer | 1 |
| 98723 | C10FD11 | Type II diabetes mellitus with hypoglycaemic coma | 2 |
| 98954 | 3883 | Diabetes treatment satisfaction questionnaire | 2 |
| 99231 | C108B11 | Type I diabetes mellitus with mononeuropathy | 1 |
| 99277 | 9NiC.00 | Did not attend DAFNE diabetes structured education programme | 1 |
| 99311 | C10E111 | Type I diabetes mellitus with ophthalmic complications | 1 |
| 99628 | Kyu0300 | [X]Glomerular disorders in diabetes mellitus | 2 |
| 99716 | C10EE12 | Insulin dependent diabetes mellitus with hypoglycaemic coma | 1 |
| 99719 | C10EA12 | Insulin-dependent diabetes without complication | 1 |
| 100033 | U60231E | [X] Adverse reaction to insulins and antidiabetic agents NOS | 2 |
| 100292 | Cyu2300 | [X]Unspecified diabetes mellitus with renal complications | 2 |
| 100347 | C10A500 | Malnutritn-relat diabetes melitus wth periph circul complctn | 2 |
| 100422 | 8HgC.00 | Discharged from diabetes shared care programme | 2 |
| 100436 | 679L000 | Education in self management of diabetes | 2 |
| 100770 | C10EF12 | Insulin dependent diabetes mellitus with diabetic cataract | 1 |
| 100964 | C10F111 | Type II diabetes mellitus with ophthalmic complications | 2 |
| 101177 | 66At.00 | Diabetic dietary review | 2 |
| 101311 | C10EC12 | Insulin dependent diabetes mellitus with polyneuropathy | 1 |
| 101455 | 9OLN.00 | Diabetes monitor invitation by SMS (short message service) | 2 |
| 101728 | 66As.00 | Diabetic on subcutaneous treatment | 2 |
| 101735 | C10E212 | Insulin-dependent diabetes mellitus with neurological comps | 1 |
| 101801 | 66At100 | Type II diabetic dietary review | 2 |
| 101881 | 2BBr.00 | Impaired vision due to diabetic retinopathy | 2 |
| 102112 | C10E611 | Type I diabetes mellitus with gangrene | 1 |
| 102163 | C10ED12 | Insulin dependent diabetes mellitus with nephropathy | 2 |
| 102201 | C10FC11 | Type II diabetes mellitus with nephropathy | 2 |
| 102434 | 66Au.00 | Diabetic erectile dysfunction review | 2 |
| 102549 | 66Aw.00 | Insulin dose | 2 |
| 102611 | 66At111 | Type 2 diabetic dietary review | 2 |
| 102620 | C10EL11 | Type I diabetes mellitus with persistent microalbuminuria | 1 |
| 102704 | 66At000 | Type I diabetic dietary review | 1 |
| 102740 | C108112 | Type 1 diabetes mellitus with ophthalmic complications | 1 |
| 102946 | C10E012 | Insulin-dependent diabetes mellitus with renal complications | 1 |
| 103761 | 671F000 | Insulin alert pat information booklet information discussed | 2 |
| 103762 | 8BAi.00 | Insulin passport completed | 2 |
| 103772 | 8CE0200 | Insulin passport given | 2 |
| 103798 | 9b92000 | Diabetic medicine | 2 |
| 103816 | 8CE0100 | Insulin alert patient information booklet given | 2 |
| 103817 | 8BAj.00 | Informed dissent not to carry insulin passport | 2 |
| 103902 | C10FG11 | Type II diabetes mellitus with arthropathy | 2 |
| 104254 | 7L10011 | Subcutaneous infusion with insulin pump | 2 |
| 104323 | C10F511 | Type II diabetes mellitus with gangrene | 2 |
| 104374 | 67D8.00 | Provision of diabetes clinical summary | 2 |
| 104453 | 66At011 | Type 1 diabetic dietary review | 1 |
| 104639 | C10FF11 | Type II diabetes mellitus with peripheral angiopathy | 2 |
| 104858 | 8BAm.00 | Insulin passport checked | 2 |
| 105302 | K08yA00 | Proteinuric diabetic nephropathy | 2 |
| 105337 | C10E811 | Type I diabetes mellitus - poor control | 1 |
| 105446 | 679c.00 | Insulin administration education | 2 |
| 105585 | 8CMW700 | Diabetes clinical pathway | 2 |
| 105740 | 2G5d.00 | O/E - Left diabetic foot at increased risk | 2 |
| 105741 | 2G5e.00 | O/E - Right diabetic foot at increased risk | 2 |
| 105784 | C109912 | Type 2 diabetes mellitus without complication | 2 |
| 106061 | C10FP11 | Type II diabetes mellitus with ketoacidotic coma | 2 |
| 106269 | 9m0..00 | Diabetic retinopathy screening administrative status | 2 |
| 106328 | 9m07.00 | Excluded diabetc retinop screen as under care ophthalmolgist | 2 |
| 106329 | 9m08.00 | Excluded from diabetic retinopathy screening as blind | 2 |
| 106332 | 9m00.00 | Eligible for diabetic retinopathy screening | 2 |
| 106360 | K27y700 | Erectile dysfunction due to diabetes mellitus | 2 |
| 106445 | 9m0E.00 | Excluded from diabetic retinopathy screen physical disorder | 2 |
| 106528 | C10FN11 | Type II diabetes mellitus with ketoacidosis | 2 |
| 106679 | 8OA3.00 | Provision of written information about diabetes and driving | 2 |
| 106722 | 9Oy0300 | Diabetic foot screening invitation second letter | 2 |
| 106723 | 9Oy0200 | Diabetic foot screening invitation first letter | 2 |
| 106738 | 9Oy0000 | Diabetic foot screening invitation | 2 |
| 106778 | 9m0C.00 | Excluded frm diabetic retinopathy screen as terminal illness | 2 |
| 107331 | 66AH100 | Conversion to insulin in secondary care | 2 |
| 107361 | 679L200 | Education about diabetes and driving | 2 |
| 107423 | 661N400 | Diabetes self-management plan review | 2 |
| 107452 | 66o..00 | Further diabetic monitoring | 2 |
| 107464 | 66AS000 | Diabetes Year of Care annual review | 2 |
| 107508 | 66AH200 | Conversion to insulin by diabetes specialist nurse | 2 |
| 107597 | 9m0D.00 | Excluded from diabetic retinopthy screen as learn disability | 2 |
| 107701 | C10FK11 | Hyperosmolar non-ketotic state in type II diabetes mellitus | 2 |
| 107739 | 679L211 | Advice about diabetes and driving | 2 |
| 107793 | 9Oy0400 | Diabetic foot screening invitation third letter | 2 |
| 107881 | K08yA11 | Clinical diabetic nephropathy | 2 |
