# Supplementary figures and images for: Health checks and cardiovascular risk factor values over six years’ follow-up: Matched cohort study using electronic health records in England
Source: PLoS Med. 2019 Jul 30;16(7):e1002863. doi: 10.1371/journal.pmed.1002863 (PMC6667114; doi:10.1371/journal.pmed.1002863)

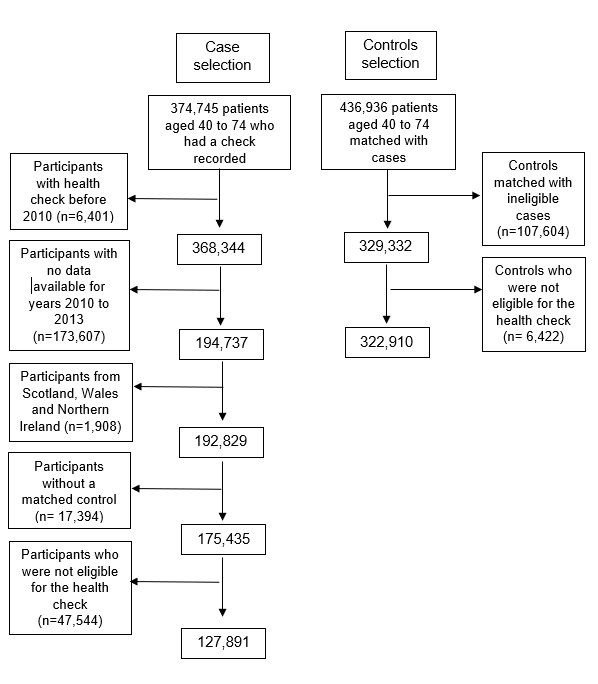


S1 Fig: Sample selection flow chart.

Supplement: S1 Fig — (DOCX) [file pmed.1002863.s003.docx]
