# Supplementary material for: Health checks and cardiovascular risk factor values over six years’ follow-up: Matched cohort study using electronic health records in England
Source: PLoS Med. 2019 Jul 30;16(7):e1002863. doi: 10.1371/journal.pmed.1002863 (PMC6667114; doi:10.1371/journal.pmed.1002863)
Supplement: S2 Fig — Red, health check participants; blue, control participants. (DOCX) [file pmed.1002863.s004.docx]

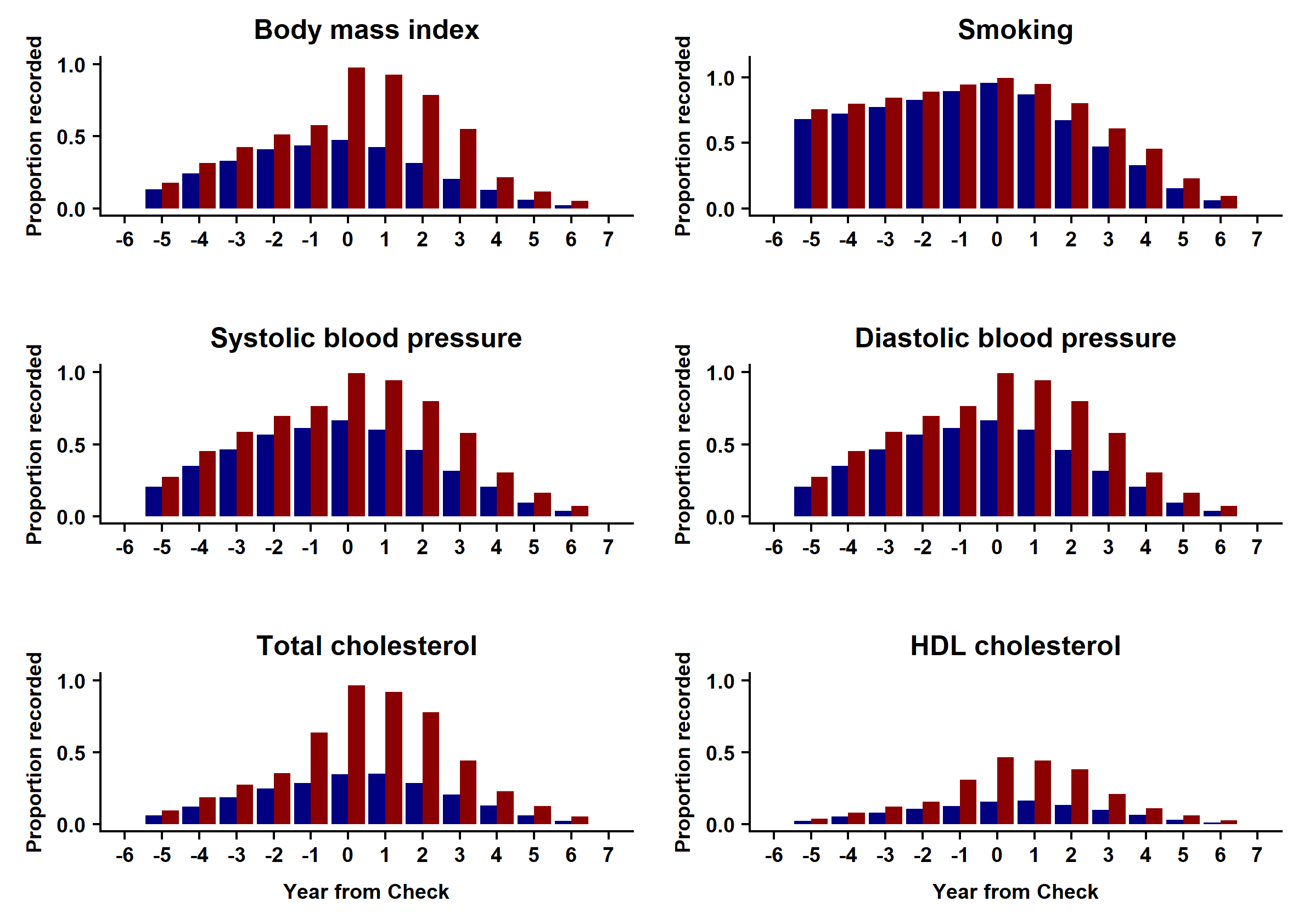


**S2 Fig: Proportion of participants with risk factor values recorded by year. Red, health check participants; blue, control participants.**
