# Supplementary material for: Health checks and cardiovascular risk factor values over six years’ follow-up: Matched cohort study using electronic health records in England
Source: PLoS Med. 2019 Jul 30;16(7):e1002863. doi: 10.1371/journal.pmed.1002863 (PMC6667114; doi:10.1371/journal.pmed.1002863)
Supplement: S3 Fig — (DOCX) [file pmed.1002863.s005.docx]

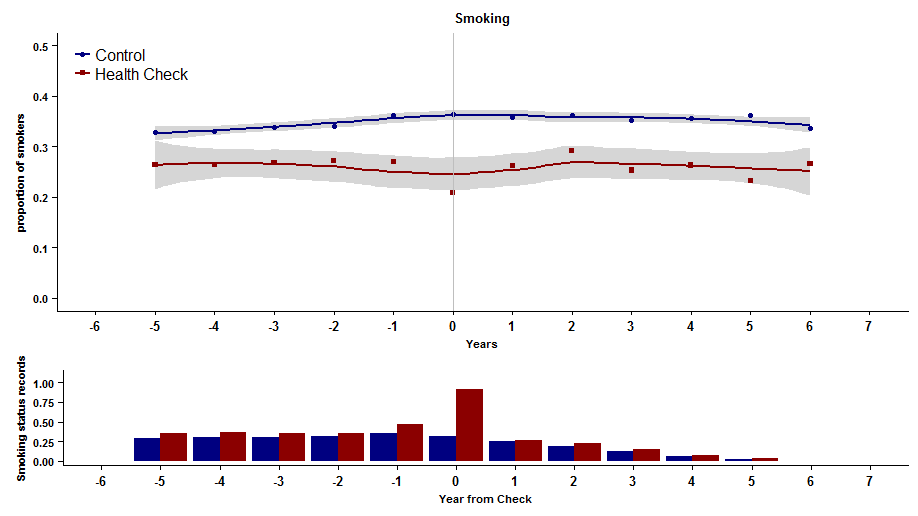

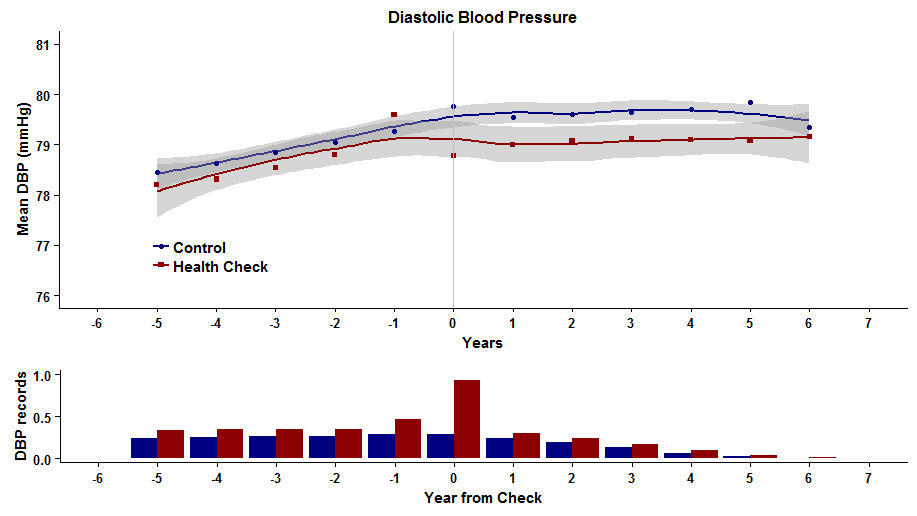

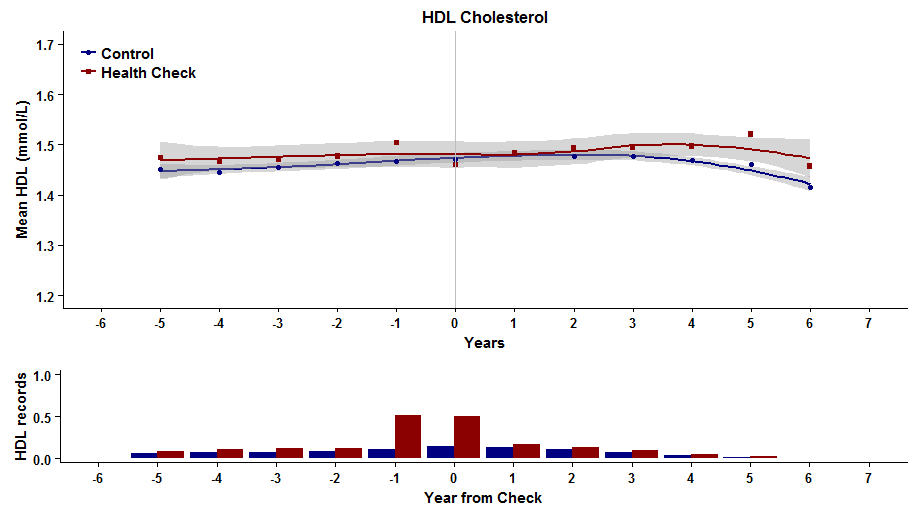

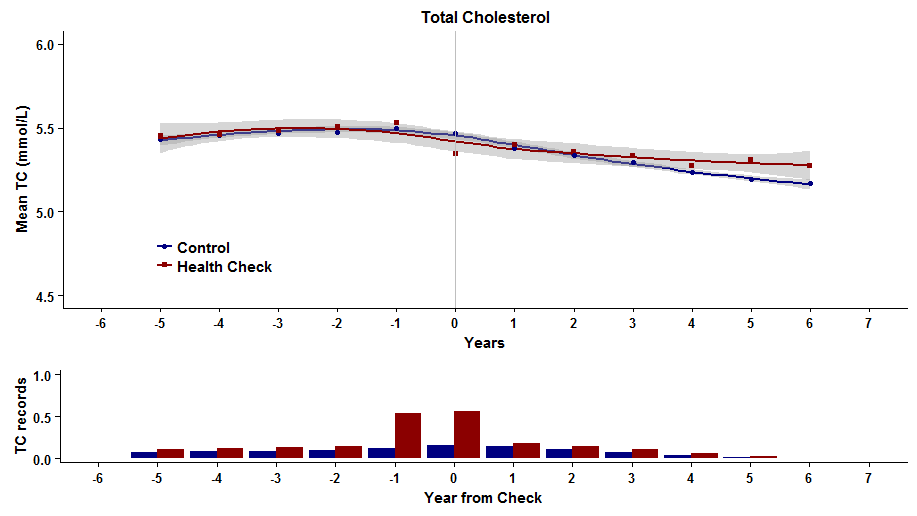

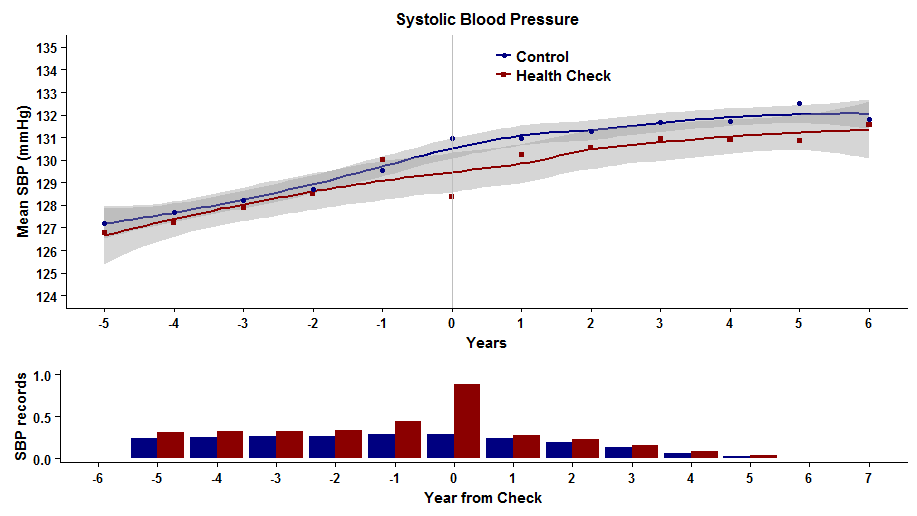

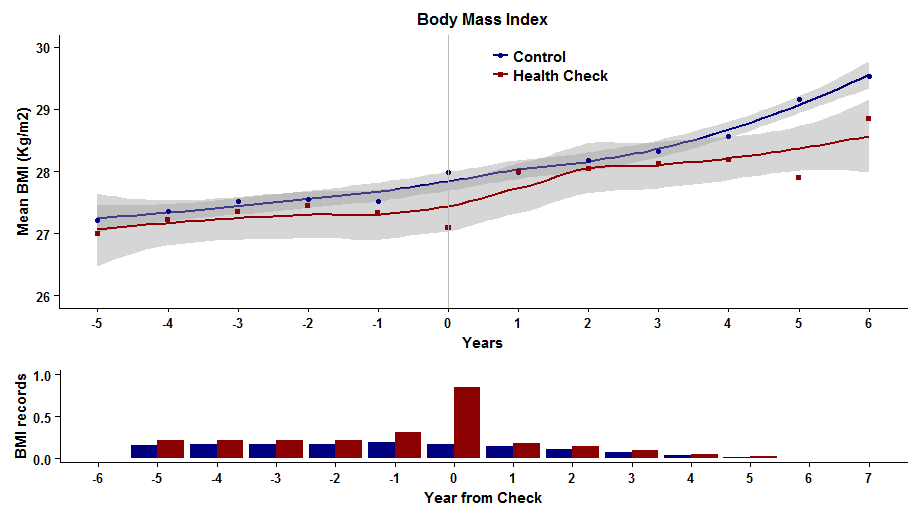


**S3 Fig: Above: Risk factors trajectories of health check and control groups five years before, and six years after, the index date. Below: Proportion of participants with risk factor recorded throughout the study period for health check and control groups.**
