# Supplementary material for: Health checks and cardiovascular risk factor values over six years’ follow-up: Matched cohort study using electronic health records in England
Source: PLoS Med. 2019 Jul 30;16(7):e1002863. doi: 10.1371/journal.pmed.1002863 (PMC6667114; doi:10.1371/journal.pmed.1002863)
Supplement: S1 Table — (DOCX) [file pmed.1002863.s006.docx]

**S1 Table: Coding of interrupted time series analysis terms.**

| Year | | Group | | Time After | |
| --- | --- | --- | --- | --- | --- |
| Health check | **Controls** | **Health check** | **Controls** | **Health check** | **Controls** |
| -5 | -5 | 1 | 0 | 0 | 0 |
| -4 | -4 | 1 | 0 | 0 | 0 |
| -3 | -3 | 1 | 0 | 0 | 0 |
| -2 | -2 | 1 | 0 | 0 | 0 |
| -1 | -1 | 1 | 0 | 0 | 0 |
| 0 | 0 | 1 | 0 | 0 | 0 |
| 1 | 1 | 1 | 0 | 1 | 0 |
| 2 | 2 | 1 | 0 | 2 | 0 |
| 3 | 3 | 1 | 0 | 3 | 0 |
| 4 | 4 | 1 | 0 | 4 | 0 |
| 5 | 5 | 1 | 0 | 5 | 0 |
| 6 | 6 | 1 | 0 | 6 | 0 |
