# Supplementary material for: Health checks and cardiovascular risk factor values over six years’ follow-up: Matched cohort study using electronic health records in England
Source: PLoS Med. 2019 Jul 30;16(7):e1002863. doi: 10.1371/journal.pmed.1002863 (PMC6667114; doi:10.1371/journal.pmed.1002863)
Supplement: S2 Table — NHS, National Health Services. (DOCX) [file pmed.1002863.s007.docx]

S2 Table: Regression analysis of the association of the NHS Health Check programme with changes in risk factors means, by sex.

|  |  | Mean difference between cases and controls | Mean change per year for cases and controls | Year following the health check | | | | | |
| --- | --- | --- | --- | --- | --- | --- | --- | --- | --- |
|  |  |  |  | **1^st^ year** | **2^nd^ year** | **3^rd^ year** | **4^th^ year** | **5^th^ year** | **6^th^ year** |
| BMI mean, Kg/m^2^ | Male | -0.09  (-0.15 to -0.03) | 0.06  (0.05 to 0.07) | -0.26  (-0.28 to -0.24) | -0.32  (-0.36 to -0.28) | -0.37  (-0.41 to -0.33) | -0.05  (-0.13 to 0.03) | -0.18  (-0.28 to -0.08) | -0.30  (-0.44 to -0.16) |
|  | Female | -0.40  (-0.46 to -0.34) | 0.08  (0.07 to 0.09) | -0.33  (-0.35 to -0.31) | -0.39  (-0.43 to -0.35) | -0.46  (-0.52 to -0.40) | -0.05  (-0.13 to 0.03) | -0.17  (-0.29 to -0.05) | -0.30  (-0.46 to -0.14) |
| SBP, mean, mm Hg | Male | -0.98  (-1.09 to -0.86) | 0.09  (0.07 to 0.11) | -1.53  (-1.63 to -1.43) | -1.69  (-1.81 to -1.57) | -1.88  (-2.01 to -1.74) | -0.93  (-1.15 to -0.71) | -1.07  (-1.34 to -0.79) | -1.20  (-1.59 to -0.81) |
|  | Female | -1.42  (-1.54 to -1.30) | 0.26  (0.24 to 0.28) | -1.65  (-1.75 to -1.55) | -1.71  (-1.83 to -1.59) | -1.91  (-2.05 to -1.77) | -0.51  (-0.71 to -0.31) | -0.82  (-1.09 to -0.55) | -1.58  (-1.95 to -1.21) |
| DBP, mean, mm Hg | Male | -0.30  (-0.38 to -0.22) | -0.09  (-0.10 to -0.08) | -0.94  (-0.99 to -0.88) | -0.98  (-1.06 to -0.90) | -0.99  (-1.09 to -0.89) | -0.81  (-0.95 to -0.67) | -0.88  (-1.06 to -0.70) | -0.91  (-1.16 to -0.66) |
|  | Female | -0.58  (-0.66 to -0.50) | -0.01  (-0.02 to -0.00) | -0.98  (-1.04 to -0.92) | -0.90  (-0.98 to -0.82) | -0.95  (-1.05 to -0.85) | -0.44  (-0.56 to -0.32) | -0.56  (-0.75 to -0.38) | -0.92  (-1.17 to -0.67) |
| TC, mean, mmol/L | Male | 0.03  (0.02 to 0.04) | -0.04  (-0.04 to -0.03) | -0.03  (-0.04 to -0.02) | -0.03  (-0.04 to -0.02) | -0.06  (-0.07 to -0.05) | -0.05  (-0.07 to -0.03) | -0.06  (-0.08 to -0.04) | -0.04  (-0.08 to -0.00) |
|  | Female | -0.01  (-0.02 to -0.00) | 0.002  (0.00 to 0.003) | -0.06  (-0.07 to -0.05) | -0.07  (-0.08 to -0.06) | -0.10  (-0.11 to -0.09) | -0.04  (-0.06 to -0.02) | -0.05  (-0.07 to -0.03) | -0.07  (-0.09 to -0.05) |
| HDL, mean, mmol/L | Male | 0.003  (-0.001 to 0.01) | 0.002  (0.001 to 0.003) | -0.004  (-0.01 to -0.002) | -0.005  (-0.01 to -0.003) | -0.01  (-0.01 to -0.006) | 0.01  (0.004 to 0.02) | 0.01  (0.002 to 0.02) | 0.02  (0.01 to 0.03) |
|  | Female | 0.02  (0.01 to 0.03) | 0.01  (0.009 to 0.01) | -0.01  (-0.01 to -0.008) | -0.01  (-0.01 to -0.006) | -0.02  (-0.02 to -0.01) | -0.01  (-0.02 to -0.004) | 0.002  (-0.01 to 0.01) | -0.01  (-0.03 to 0.01) |
| Smoking, Odds ratio | Male | 0.70  (0.69 to 0.72) | 0.97  (0.96 to 0.97) | 0.96  (0.95 to 0.97) | 0.92  (0.91 to 0.94) | 0.91  (0.89 to 0.93) | 0.91  (0.89 to 0.93) | 0.92  (0.87 to 0.96) | 0.89  (0.84 to 0.94) |
|  | Female | 0.69  (0.68 to 0.71) | 0.97  (0.96 to 0.97) | 0.98  (0.97 to 0.99) | 0.94  (0.93 to 0.96) | 0.91  (0.89 to 0.93) | 0.92  (0.91 to 0.94) | 0.92  (0.98 to 0.96) | 0.91  (0.86 to 0.97) |

BMI, body mass index; SBP, systolic blood pressure; DBP, diastolic blood pressure; TC, total cholesterol; HDL, high density lipoprotein.

Differences were estimated as cases-controls using generalised estimation equation models adjusting for each variable shown as well as age and deprivation quintile.
