# Supplementary material for: Health checks and cardiovascular risk factor values over six years’ follow-up: Matched cohort study using electronic health records in England
Source: PLoS Med. 2019 Jul 30;16(7):e1002863. doi: 10.1371/journal.pmed.1002863 (PMC6667114; doi:10.1371/journal.pmed.1002863)
Supplement: S3 Table — Figures are adjusted mean differences (95% confidence interval) except where indicated. (DOCX) [file pmed.1002863.s008.docx]

S3 Table: Interrupted time series analysis of one year carried forward comparing health check and control participants. Figures are adjusted mean differences (95% confidence interval) except where indicated.

|  | Mean difference between cases and controls | Mean change per year for cases and controls | Year following the health check | | | | | |
| --- | --- | --- | --- | --- | --- | --- | --- | --- |
|  |  |  | **1^st^ year** | **2^nd^ year** | **3^rd^ year** | **4^th^ year** | **5^th^ year** | **6^th^ year** |
| BMI mean, Kg/m^2^ | -0.37  (-0.41 to -0.33) | 0.10  (0.09 to 0.10) | -0.26  (-0.28 to -0.24) | 0.15  (0.12 to 0.19) | 0.12  (0.06 to 0.18) | 0.07  (-0.01 to 0.15) | -0.12  (-0.22 to -0.02) | -0.36  (-0.52 to -0.20) |
| Current smoking, Odds ratio | 0.61  (0.59 to 0.62) | 0.99  (0.99 to 1.00) | 0.97  (0.96 to 0.98) | 1.07  (1.06 to 1.08) | 1.07  (1.06 to 1.08) | 1.05  (1.03 to 1.07) | 1.05  (1.03 to 1.07) | 1.02  (0.98 to 1.06) |
| SBP, mean, mm Hg | -1.46  (-1.54 to -1.38) | 0.38  (0.36 to 0.40) | -1.07  (-1.15 to -0.99) | 0.08  (-0.04 to 0.20) | -0.02  (-0.16 to 0.12) | -0.11  (-0.29 to 0.07) | -0.41  (-0.65 to -0.17) | -0.60  (-0.97 to -0.23) |
| DBP, mean, mm Hg | -0.55  (-0.61 to -0.49) | 0.07  (0.06 to 0.08) | -0.47  (-0.51 to -0.43) | -0.23  (-0.31 to -0.15) | -0.25  (-0.35 to -0.15) | -0.33  (-0.45 to -0.21) | -0.47  (-0.63 to -0.31) | -0.47  (-0.71 to -0.23) |
| TC, mean, mmol/L | 0.01  (0.002 to 0.02) | -0.02  (-0.02 to -0.01) | -0.07  (-0.08 to -0.06) | -0.04  (-0.05 to -0.03) | -0.04  (-0.05 to -0.03) | -0.05  (-0.06 to -0.04) | -0.05  (-0.07 to -0.03) | 0.001  (-0.04 to 0.04) |
| HDL, mean, mmol/L | 0.01  (0.006 to 0.01) | 0.01  (0.009 to 0.01) | -0.01  (-0.01 to -0.008) | 0.003  (-0.001 to 0.007) | 0.002  (-0.002 to 0.01) | 0.002  (-0.004 to 0.01) | 0.01  (0.002 to 0.02) | 0.01  (-0.002 to 0.02) |

BMI, body mass index; SBP, systolic blood pressure; DBP, diastolic blood pressure; TC, total cholesterol; HDL, high density lipoprotein.

Differences were estimated as cases-controls using generalised estimation equation models adjusting for each variable shown as well as age, sex and deprivation quintile.
