# Supplementary material for: Health checks and cardiovascular risk factor values over six years’ follow-up: Matched cohort study using electronic health records in England
Source: PLoS Med. 2019 Jul 30;16(7):e1002863. doi: 10.1371/journal.pmed.1002863 (PMC6667114; doi:10.1371/journal.pmed.1002863)
Supplement: S4 Table — Figures are adjusted mean differences (95% confidence interval) except where indicated. ITS, interrupted-time series. (DOCX) [file pmed.1002863.s009.docx]

S4 Table: Interrupted time series analysis of recorded data comparing health check and control participants. Figures are adjusted mean differences (95% confidence interval) except where indicated.

|  | Mean difference between cases and controls | Mean change per year for cases and controls | Year following the health check | | | | | |
| --- | --- | --- | --- | --- | --- | --- | --- | --- |
|  |  |  | **1^st^ year** | **2^nd^ year** | **3^rd^ year** | **4^th^ year** | **5^th^ year** | **6^th^ year** |
| BMI mean, Kg/m^2^ | -0.41  (-0.45 to -0.37) | 0.10  (0.09 to 0.11) | 0.28  (0.22 to 0.34) | 0.24  (0.18 to 0.30) | 0.22  (0.14 to 0.30) | 0.11  (0.01 to 0.23) | -0.29  (-0.45 to -0.13) | -0.03  (-0.40 to 0.34) |
| Current smoking, Odds ratio | 0.62  (0.61 to 0.63) | 0.99  (0.99 to 0.99) | 1.01  (0.99 to 1.03) | 1.12  (1.09 to 1.14) | 0.97  (0.95 to 0.99) | 0.99  (0.95 to 1.02) | 0.88  (0.83 to 0.93) | 1.04  (0.89 to 1.21) |
| SBP, mean, mm Hg | -1.51  (-1.59 to -1.43) | 0.42  (0.40 to 0.44) | 0.32  (0.18 to 0.46) | 0.14  (-0.02 to 0.30) | 0.06  (-0.12 to 0.24) | -0.36  (-0.61 to -0.11) | -0.66  (-1.03 to -0.29) | -0.50  (-1.38 to 0.38) |
| DBP, mean, mm Hg | -0.53  (-0.59 to -0.47) | 0.09  (0.08 to 0.10) | -0.17  (-0.25 to -0.09) | -0.22  (-0.32 to -0.12) | -0.31  (-0.43 to -0.19) | -0.57  (-0.73 to -0.41) | -0.62  (-0.86 to -0.38) | -0.69  (-1.26 to -0.12) |
| TC, mean, mmol/L | 0.005  (-0.003 to 0.01) | -0.02  (-0.02 to -0.01) | -0.02  (-0.03 to -0.01) | -0.04  (-0.05 to -0.03) | -0.05  (-0.06 to -0.03) | -0.07  (-0.09 to -0.05) | -0.01  (-0.05 to 0.03) | 0.01  (-0.07 to 0.09) |
| HDL, mean, mmol/L | 0.01  (0.008 to 0.01) | 0.01  (0.009 to 0.01) | 0.02  (0.01 to 0.02) | 0.02  (0.01 to 0.02) | 0.02  (0.01 to 0.02) | 0.02  (0.01 to 0.03) | 0.02  (0.01 to 0.02) | 0.01  (-0.01 to 0.03) |

BMI, body mass index; SBP, systolic blood pressure; DBP, diastolic blood pressure; TC, total cholesterol; HDL, high density lipoprotein.

Differences were estimated as cases-controls using generalised estimation equation models adjusting for each variable shown as well as age, sex and deprivation quintile.
