# Supplementary material for: Health checks and cardiovascular risk factor values over six years’ follow-up: Matched cohort study using electronic health records in England
Source: PLoS Med. 2019 Jul 30;16(7):e1002863. doi: 10.1371/journal.pmed.1002863 (PMC6667114; doi:10.1371/journal.pmed.1002863)
Supplement: S5 Table — Sensitivity analysis with one control per case. Figures are adjusted mean differences (95% confidence interval) except where indicated. ITS, interrupted-time series. (DOCX) [file pmed.1002863.s010.docx]

S5 Table: Interrupted time series analysis comparing health check and control participants. Sensitivity analysis with one control per case. Figures are adjusted mean differences (95% confidence interval) except where indicated.

|  | Mean difference between cases and controls | Mean change per year for cases and controls | Year following the health check | | | | | |
| --- | --- | --- | --- | --- | --- | --- | --- | --- |
|  |  |  | **1^st^ year** | **2^nd^ year** | **3^rd^ year** | **4^th^ year** | **5^th^ year** | **6^th^ year** |
|  |  |  |  |  |  |  |  |  |
| Body mass index  mean, Kg/m^2^ | -0.37  (-0.42 to -0.33) | 0.06  (0.06 to 0.07) | -0.23  (-0.25 to -0.21) | -0.27  (-0.29 to -0.24) | -0.32  (-0.36 to -0.29) | -0.05  (-0.10 to 0.008) | -0.15  (-0.22 to -0.08) | -0.26  (-0.36 to -0.16) |
|  |  |  |  |  |  |  |  |  |
| Current smoking, Odds ratio | 0.71  (0.69 to 0.71) | 0.97  0.96 to 0.97) | 0.98  (0.97 to 0.99) | 0.94  (0.93 to 0.95) | 0.92  (0.91 to 0.94) | 0.93  (0.91 to 0.95) | 0.94  (0.91 to 0.95) | 0.92  (0.88 to 0.96) |
|  |  |  |  |  |  |  |  |  |
| Systolic BP,  mean, mm Hg | -1.64  (-1.74 to -1.54) | 0.15  (0.14 to 0.17) | -1.51  (-1.58 to -1.45) | -1.60  (-1.68 to -1.51) | -1.79  (-1.90 to -1.69) | -0.64  (-0.79 to -0.49) | -0.88  (-1.08 to -0.68) | -1.30  (-1.58 to -1.02) |
|  |  |  |  |  |  |  |  |  |
| Diastolic BP, mean, mm Hg | -0.67  (-0.74 to -0.61) | -0.06  (-0.07 to -0.05) | -0.92  (-0.96 to -0.88) | -0.88  (-0.94 to -0.83) | -0.90  (-0.97 to -0.83) | -0.56  (-0.66 to -0.46) | -0.67  (-0.80 to -0.54) | -0.85  (-1.02 to -0.67) |
|  |  |  |  |  |  |  |  |  |
| Total cholesterol, mean, mmol/L | 0.01  (-0.003 to 0.01) | -0.02  (-0.02 to -0.02) | -0.04  (-0.05 to -0.04) | -0.05  (-0.06 to -0.05) | -0.08  (-0.09 to -0.07) | -0.05  (-0.07 to -0.04) | -0.06  (-0.08 to -0.05) | -0.06  (-0.08 to -0.04) |
|  |  |  |  |  |  |  |  |  |
| HDL cholesterol, mean, mmol/L | 0.01  (0.007 to 0.01) | 0.004  (0.004 to 0.005) | -0.006  (-0.008 to -0.004) | -0.01  (-0.01 to -0.008) | -0.01  (-0.02 to -0.01) | 0.003  (-0.002 to 0.007) | 0.009  (0.003 to 0.015) | 0.006  (-0.002 to 0.014) |
|  |  |  |  |  |  |  |  |  |

Differences were estimated as cases-controls using generalised estimation equation models adjusting for each variable shown as well as age, sex and deprivation fifth.
